# Can salivary and skin microbiome become a biodetector for aging-associated diseases? Current insights and future perspectives

**DOI:** 10.3389/fragi.2024.1462569

**Published:** 2024-10-17

**Authors:** Fahrul Nurkolis, Trianna Wahyu Utami, Aiman Idrus Alatas, Danar Wicaksono, Rudy Kurniawan, Satria Rafi Ratmandhika, Kartika Taufani Sukarno, Yehezkiel Gian Pradipta Pahu, Bonglee Kim, Trina Ekawati Tallei, Raymond Rubianto Tjandrawinata, Ananto Ali Alhasyimi, Reggie Surya, Helen Helen, Princella Halim, Adi Muradi Muhar, Rony Abdi Syahputra

**Affiliations:** ^1^ Department of Biological Sciences, Faculty of Sciences and Technology, State Islamic University of Sunan Kalijaga (UIN Sunan Kalijaga), Yogyakarta, Indonesia; ^2^ Department of Dental Biomedical Sciences, Faculty of Dentistry, Universitas Gadjah Mada, Yogyakarta, Indonesia; ^3^ Program of Clinical Microbiology Residency, Faculty of Medicine, Universitas Indonesia, Jakarta, Indonesia; ^4^ Alumnus Department of Dermatology and Venereology, Faculty of Medicine, Public Health, and Nursing, Universitas Gadjah Mada, Yogyakarta, Indonesia; ^5^ Graduate School of Medicine, Faculty of Medicine, Hasanuddin University, Makassar, Indonesia; ^6^ Medical School Department, Faculty of Medicine, Brawijaya University, Malang, Indonesia; ^7^ Department of Pathology, College of Korean Medicine, Kyung Hee University, Seoul, Republic of Korea; ^8^ Department of Biology, Faculty of Mathematics and Natural Sciences, Sam Ratulangi University, Manado, Indonesia; ^9^ Department of Biotechnology, Faculty of Biotechnology, Atma Jaya Catholic University of Indonesia, Jakarta, Indonesia; ^10^ Department of Orthodontics, Faculty of Dentistry, Universitas Gadjah Mada, Yogyakarta, Indonesia; ^11^ Department of Food Technology, Faculty of Engineering, Bina Nusantara University, Jakarta, Indonesia; ^12^ Department of Pharmacology, Faculty of Pharmacy, Universitas Sumatera Utara, Medan, Indonesia; ^13^ Faculty of Medicine, Universitas Sumatera Utara, Medan, Indonesia

**Keywords:** oral microbiome, skin microbiome, age-related disease, biodetector, aging

## Abstract

Growth and aging are fundamental elements of human development. Aging is defined by a decrease in physiological activities and higher illness vulnerability. Affected by lifestyle, environmental, and hereditary elements, aging results in disorders including cardiovascular, musculoskeletal, and neurological diseases, which accounted for 16.1 million worldwide deaths in 2019. Stress-induced cellular senescence, caused by DNA damage, can reduce tissue regeneration and repair, promoting aging. The root cause of many age-related disorders is inflammation, encouraged by the senescence-associated secretory phenotype (SASP). Aging’s metabolic changes and declining immune systems raise illness risk via promoting microbiome diversity. Stable, individual-specific skin and oral microbiomes are essential for both health and disease since dysbiosis is linked with periodontitis and eczema. Present from birth to death, the human microbiome, under the influence of diet and lifestyle, interacts symbiotically with the body. Poor dental health has been linked to Alzheimer’s and Parkinson’s diseases since oral microorganisms and systemic diseases have important interactions. Emphasizing the importance of microbiome health across the lifetime, this study reviews the understanding of the microbiome’s role in aging-related diseases that can direct novel diagnosis and treatment approaches.

## 1 Introduction

The growth and aging processes are fundamental aspects of human development that occur throughout the lifespan. The aging process is a complex biological phenomenon characterized by the gradual decline in physiological function and increased susceptibility to disease and death over time. While aging is a natural part of life, it is influenced by a combination of genetic, environmental, and lifestyle factors (Jang, 2020). Aging eventually leads to age-related disease, such as cardiovascular disease, musculoskeletal disease, neurodegenerative disease, and other organ disease. Total global death of aging associated disease in 1990 until 2019 is 16.1 million global death (69,2%) with the top 10 highest disease, include ischemic heart disease 5.0 million; stroke 3.8 million; chronic obstructive pulmonary disease (COPD) 2.2 million; Alzheimer’s disease 1.0 million; lower respiratory infections 1.0 million; tracheal, bronchus, and lung cancer 0.8 million; diarrheal disease 0.7 million; hypertesive heart disease 0.6 million; diabetes melitus 0.6 million; and chronic kidney disease 0.5 million (Li et al., 2023).

**FIGURE 1:**
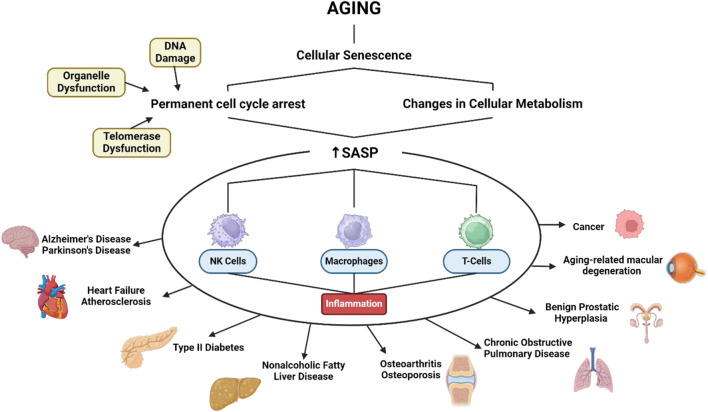
SASP related to various age-related diseases. Created with BioRender.com Premium License by Fahrul Nurkolis (https://app.biorender.com, accessed on 10th July 2024).

The mechanisms of aging involve various biological pathways (Figure 1), including celullar senescence. This can contribute to aging by reducing the ability of tissues to regenerate and repair themselves (Li et al., 2021). Senescence is characterized by a permanent cell cycle arrest in response to various cellular stresses, including DNA damage, telomere dysfunction, and organelle dysfunction. This arrest is accompanied by changes in cellular metabolism, including the production and secretion of a complex array of factors known as senescence-associated secretory phenotype (SASP) (Liao et al., 2021). Once SASP is formed it is irreversible in most senescent cells. This suggests that SASP is a more persistent feature of aging. One of the primary roles of the SASP is to communicate with various immune cells, including natural killer (NK) cells, macrophages, and T cells to facilitate the removal of old cells. In conditions when immune senescence causes inflammation, a response that is often considered dangerous and it can be the root cause of most diseases in middle-aged and elderly people (Zhao et al., 2024).

A decrease in the function of the immune system and metabolism in the aging process causes an increase in risk factors for the growth of microbiome diversity in the body, including bacteria, viruses, archae and fungi which have clinical manifestations in disease in the human body. Skin and oral microbiomes are highly individual and relatively stable over time. Each individual has a specific composition of microorganisms in each environment and relatively consistent throughout life (Lovisolo et al., 2022). There is great individual variability in the composition of the skin and oral microbiome. The variability between identical twins is almost the same as non-identical twins or the average variability from person to person. The skin identification location is divided into three areas, include sebaceous; moist; and dry areas. Each area of skin has a unique diversity of microbes. Like the skin microbiome, the oral microbiome is also divided into 3 areas including the buccal mucosa, gingiva, and hard palate; the saliva, tongue, tonsils, and throat; and the supra- and subgingival plaque. This area is based on the similarity of the bacterial community in an individual, if take samples from several locations in one group, it can see consistency in the composition of the microbial species level (Buerger, 2020a). Skin microbiome has been shown to be a better predictor of chronological age. Skin microbiome has ability to detect aging-related changes in the skin, such as dryness, collagen fragmentation, and sebum secretion (Larson et al., 2022). The oral microbiome has been linked to various age-related disease including periodontitis, which is a significant risk factor for cardiovascular disease, diabetes, and other systemic condition.

An imbalance in the microbiome of the skin or oral is associated with the body’s pathological condition and the presence of a disease or infection. The microbiome also has an influence on every human life that overlaps with aging. Therefore, further discussion is needed regarding insights and future perspectives regarding the use of skin/oral microbiome as a disease detector related to Aging-associated diseases. This is also supported by the absence of other authors who have conducted review articles related to this potential and reviewed the potential of the skin and oral microbiome has a relatively stable throughout life so it can be potential used to detect diseases caused by aging. Therefore, this review article aims to complement current findings and answer existing gaps by trying to create a bridge between Aging-associated diseases and the skin and oral microbiome, so that it can provide the latest insights in the management of Aging-associated diseases, which have never been reported before.

This paper exploring the potential of the salivary and skin microbiome as biodetectors for aging-associated diseases presents a fascinating and novel approach to understanding health biomarkers. By investigating the unique microbial communities found in saliva and on the skin, the research opens up new avenues for early detection and monitoring of age-related conditions. This innovative perspective not only highlights the intricate relationship between our microbiome and overall health but also suggests that these microbial signatures could serve as non-invasive indicators for assessing disease risk. Such insights could revolutionize preventative healthcare strategies, making them more accessible and personalized, ultimately enhancing our ability to combat aging-related illnesses.

## 2 Microbiome in general

The human microbiome refers to genomic content of microbiota that reside in spesific anatomical sites. Microbiota itself refer to a set of microorganism that live all around human body, comprising bacteria, archaea, eukaryotes, or even viruses. These microorganism not only live in human body but also interact with each other and our body creating a distinct ecosystem that adapted to the spesific anatomical site. For example, microbiota residing in gastrointestinal tract are facultative anareob, while strict aerobes tend to live on the skin and in the respiratory tract. The human microbiome interaction can manifest as mutualistic, parasitic, or commensal relationships (Ogunrinola et al., 2020). The human microbiome exist since the begining of childbirth until death, maintaining symbiotic interactions with the human body under physiological conditions. Throughout a person’s lifetime. The microbiome undergoes continuous evolution in response to changes in the human body (Ogunrinola et al., 2020). Lifestyle factors, hormonal fluctuations, nutrition, antimicrobial consumption, underlying diseases, and aging all contribute to these changes (Ogunrinola et al., 2020; Rackaityte and Lynch, 2020). Such alterations can shift the balance of interactions between the human body and its microbiota from symbiosis to dysbiosis, thereby predisposing individuals to various diseases (Ogunrinola et al., 2020). While human microbiome research has advanced significantly, several limitations persist. The diversity and complexity of the microbiome across individuals and anatomical sites hinder generalized conclusions. Much of the current research is correlational, complicating the establishment of causative links between microbiome changes and health outcomes. Technological constraints, such as incomplete microbial diversity capture by current sequencing methods, further limit the field. Additionally, many studies are conducted on small, homogenous populations, reducing global applicability. Lastly, uncontrollable environmental factors and lifestyle variations significantly impact microbiome composition and function, adding complexity to research outcomes.

### 2.1 Oral/salivary microbiome in general

The oral cavity and saliva are recognized as habitats for various microorganisms to reside and proliferate (Polak and Shapira, 2018). Furthermore, studies have demonstrated that microorganisms present in the oral cavity differ from those found in other parts of the body (Costello et al., 2009). Additionally, microorganisms residing in the oral cavity are believed to possess the ability to selectively adhere to oral mucosa rather than other body parts, resulting in a distinct microbial community compared to other body regions (Long and Swenson, 1976). Moreover, saliva contains essential components such as proteins, carbohydrates, and enzymes, which play a crucial role in maintaining the ecological environment of the oral cavity (Belstrøm, 2020). An example of this is during tooth development, where saliva provides nutritional intake for the protective layer of tooth growth known as dental plaque (Jakubovics et al., 2000).

Generally, the oral microbiome present in children consists of gram-positive bacteria such as *Streptococcus mutans, Streptococcus sanguinis, Actinomyces spp, Lactobacillus*, and *Rothia*, as well as gram-negative bacteria including nonpigmenting *Prevotella spp, Porphyromonas spp, Neisseria*, and *Capnocytophaga*. Subsequently, upon reaching puberty, bacteria such as *Spirochaetes, Veillonella, Prevotella*, and black-pigmented *Bacteroides* (e.g., *Bacteroides intermedius*) become more readily identifiable and prevalent (Samaranayake and Matsubara, 2017). Apart from bacterial populations, there also exists a fungal population as part of the oral microbiome, albeit significantly less abundant compared to bacteria, estimated to be only <0.1% in the oral cavity. Common fungal species found in the mouth include species from the genera *Candida, Aspergillus, Penicillium, Schizophyllum, Rhodotorula,* and *Gibberella* (Willis and Gabaldón, 2020).

When minor and short-term disturbances occur in the components within the oral cavity due to internal or external stressors, the oral microbiome generally remains unchanged. However, if these disturbances are significant or prolonged, changes in the oral microbiome can occur (Belstrøm, 2020). Such changes can lead to diseases such as periodontitis, gingivitis, dental caries, oral cancer, and esophageal cancer. An example of this is the presence of *Porphyromonas gingivalis* and *Treponema denticola*, which are closely associated with periodontitis (Willis and Gabaldón, 2020).

Several studies have indicated that dietary changes can lead to alterations in the oral microbiome (Adler et al., 2016; Shoer et al., 2023). With the progression of time, industrialization, agriculture, and global trends, there have been numerous dietary changes, ranging from vegetarian to carnivorous diets, high-glycemic diets, and high dairy product diets. Consequently, there is also a pathological alteration in the composition and community of the oral microbiome, including an increase in acid-producing bacteria populations and acid-tolerant species (Sedghi et al., 2000). Furthermore, lifestyle changes, such as decreased frequency of toothbrushing, can increase the occurrence of dental plaque and gingivitis (Ccahuana-Vasquez et al., 2019). Moreover, the use of mouthwash can reduce the presence of one oral pathogen, *Neisseria gonorrhoeae* (Chow et al., 2017).

### 2.2 Skin microbiome in general

The skin, being the largest organ of the human body, serves as a crucial barrier against the external environment. Continuously exposed to the outside world, the skin, with its numerous invaginations and folds, provides favorable environments for various microorganisms. Consequently, the human skin microbiome exhibits high diversity, encompassing microorganisms such as bacteria, fungi, viruses, and mites (Grice and Segre, 2011). Among these, bacteria are the most prevalent, whereas fungi are comparatively less common. While the majority of skin microbes are commensal, in rare instances, they have been implicated in disease (Buerger, 2020). In terms of bacterial density within the human body, the skin ranks second only to the gut, harboring approximately 10^^4^ to 10^^8^ bacteria per cm^2^ on its surface (Smythe and Wilkinson, 2023).

The microorganisms inhabiting the skin play a crucial role in its function as a barrier. Their presence ensures that more harmful microorganisms are prevented from colonizing and invading the skin. For instance, *Corynebacterium accolens* can modulate the microenvironment by producing free fatty acids with antibacterial properties. These free fatty acids can inhibit opportunistic pathogens such as *Streptococcus pneumoniae*. Additionally, it is suggested that microorganisms may educate the abundant T cells found in the skin to recognize pathogens (Smythe and Wilkinson, 2023). Given these functions, any alteration leading to dysbiosis of the skin microbiome is closely associated with various skin diseases such as eczema, acne, and chronic wounds chronic (Byrd et al., 2018).

The skin can be physiologically characterized into three types: sebaceous or oily, moist, and dry. These three distinct habitats give rise to specific compositions of microorganisms (Byrd et al., 2018). Factors such as exposure to ultraviolet radiation, moisture levels, sebum production, pH balance, oxygen availability, and temperature are influential in determining the types of bacteria inhabiting the skin (Smythe and Wilkinson, 2023).

In oily areas, bacteria must possess the capability to metabolize lipids and exhibit tolerance to the acidity resulting from high concentrations of fatty acids. *Cutibacterium spp*., previously identified as *Propionibacterium*, fulfill these requirements owing to their possession of lipases capable of breaking down triglycerides into propionic acid. Consequently, these bacteria dominate the sebaceous skin microbiota, with *Staphylococcus spp*., including *S. aureus* and *S. epidermidis*, following as the second most prevalent species (Smythe and Wilkinson, 2023; Byrd et al., 2018).

In moist skin areas like the antecubital and popliteal fossae, *Staphylococcus* and *Corynebacterium* are the most dominant organisms (Byrd et al., 2018). *Staphylococcus*, being halotolerant, is frequently encountered in sweaty and moist regions, whereas *Corynebacterium* thrives in warm and humid environments (Smythe and Wilkinson, 2023).

In dry skin areas like the palms and soles, microbial diversity is higher and its composition is relatively less stable compared to other areas of the skin. This discrepancy yields different results in studies; for instance, Grice et al. discovered Flavobacteriales and Beta-Proteobacteria as predominant, whereas in Oh et al. study, *Cutibacterium* emerged as the most dominant. Another characteristic of dry skin is the presence of numerous gram-negative organisms, which are typically rare colonizers of the skin (Grice and Segre, 2011).

Unlike bacteria, fungi colonize the human skin irrespective of physiological characteristics. On the torso and upper extremities, *Malassezia* dominates the fungal proportion. However, on the feet, fungal diversity increases, comprising various species such as *Malassezia, Cryptococcus, Rhodotorula, Epicoccum,* and *Aspergillus* (Byrd et al., 2018).

The human microbiome in the skin generally maintains relative stability over 2 years, as observed in studies. However, throughout various life stages, the skin microbiome undergoes continuous modifications in response to changing skin conditions. In prepubescent children, the skin microbiome is characterized by a high abundance of Firmicutes, Bacteroidetes, and Proteobacteria, along with a diverse fungal population. Upon reaching puberty, hormonal shifts trigger increased activity of the sebaceous glands, promoting the growth of lipophilic organisms such as Corynebacterium spp. and Cutibacterium spp (Byrd et al., 2018).

**FIGURE 2:**
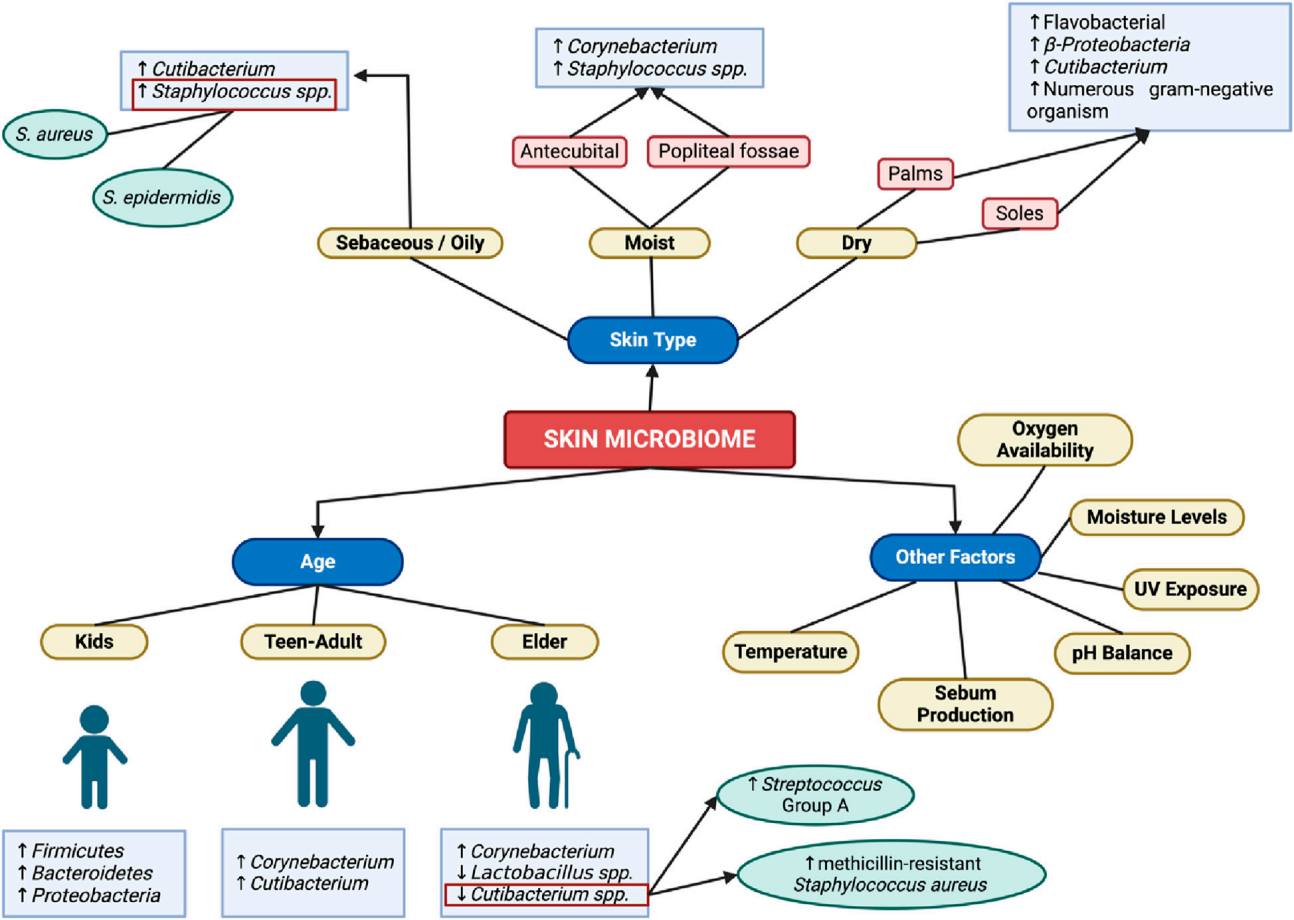
Skin Microbiome in General. Created with BioRender.com Premium License by Fahrul Nurkolis (https://app.biorender.com, accessed on 10th July 2024).

As individuals age, there is a rise in Corynebacterium levels and a decline in *Cutibacterium* and *Lactobacillus* populations (Figure 2). This shift can lead to dysbiosis and colonization by pathogenic bacteria. For instance, the decrease in *Cutibacterium*, known for its production of free fatty acids and thiopeptide antibiotics, results in reduced inhibition of the growth of *Streptococcus* group A and methicillin-resistant *Staphylococcus aureus* (Smythe and Wilkinson, 2023).

## 3 Pathogenic and regulatory mechanisms of aging-related diseases

### 3.1 Alzheimer’s disease (AD)

Alzheimer’s disease is one of the most common types of dementia, affecting two-thirds of dementia cases in individuals aged 65 and older. It is a progressive neurodegenerative disorder that gradually leads to impairments in both motor and cognitive functions (Kumar et al., 2018). Alzheimer’s disease is categorized into three stages based on the severity of symptoms, namely pre-symptomatic, mild, and dementia stages. A prevalent early symptom of Alzheimer’s disease is episodic short-term memory loss without impairment of long-term memory. These episodes of short-term memory loss may be accompanied by difficulties in problem-solving, decision-making, executive functioning impairment, decreased motivation, and disorganization (Maccioni et al., 2018; Tang et al., 2019; Zilberzwige-Tal and Gazit, 2018).

Alzheimer’s disease is characterized by neuronal death that initiates in the entorhinal cortex of the hippocampus. Additionally, there is abnormal accumulation of neuritic plaques and neurofibrillary tangles. Risk factors for Alzheimer’s disease encompass various factors, including head trauma, depression, genetic predisposition, cardiovascular disease, smoking behavior, and family history. Moreover, advancing age stands as the most dominant risk factor in Alzheimer’s disease pathogenesis (Nicolas et al., 2018; Liljegren et al., 2018; Tong et al., 2018). This is evidenced by a 10% increase in Alzheimer’s disease prevalence at the age of 65 and a 40% increase at the age of 85 (Kumar et al., 2018).

One condition that can be observed in the aging process is alterations in DNA methylation. In advanced age, DNA methylation processes can shift towards extreme methylation or demethylation, resulting in products with either more methylated or less methylated DNA (Heyn et al., 2012). These changes in DNA methylation processes may serve as one of the contributing factors to Alzheimer’s disease, as they are suspected to disrupt specific genomic regions, such as ankyrin 1 (ANK1) or 5hmC, which are associated with the onset of Alzheimer’s disease (Lunnon et al., 2014; De Jager et al., 2014; Zhao et al., 2017). This could potentially serve as a novel therapeutic approach for Alzheimer’s disease in the future.

Histones also play a role in Alzheimer’s disease in the context of aging (Lardenoije et al., 2015). In aging conditions, changes in histone modifications occur, particularly a decrease in histone acetylation processes, as observed by the loss of heterochromatin and specific histone marks, such as H1 tail, H3, and H4 (O Sullivan et al., 2010; Feser et al., 2010; Funayama et al., 2006). Two studies indicate that when histone acetylation processes are enhanced, cognitive improvement is observed in aging and Alzheimer’s disease mouse models (Kilgore et al., 2010; Benito et al., 2015). These studies are further supported by other research which suggests that dysregulation of H4K12ac can lead to cognitive impairment in aging rodent models (Peleg et al., 2010).

Furthermore, aging and Alzheimer’s disease are intertwined in the realm of RNA. In rodent animal models, the presence of microRNAs (miRNAs) indicates neuronal loss and decreased mobility (Junn and Mouradian, 2012). Additionally, certain miRNAs have the potential to serve as biomarkers for Alzheimer’s disease (Millan, 2017; Tan et al., 2013). Moreover, long noncoding RNAs (lncRNAs) also exhibit connections with the processes of senescence and aging (Kour and Rath, 2016). The process of RNA editing also plays a role in Alzheimer’s disease. Reduced RNA editing processes have been observed in specific brain areas, including the hippocampus (Khermesh et al., 2016).

In addition, the neurogenic capacity of neural stem cells also plays a significant role in Alzheimer’s disease. In the aging process, there is a decrease in neurogenesis capacity in the central nervous system (Fan et al., 2017; Montaron et al., 1999). This may lead to cognitive decline conditions by contributing to neural damage (Devanand et al., 2000). Moreover, a study in an Alzheimer’s disease rodent model showed that neural stem cell transplantation could slow the progression of Alzheimer’s disease (Blurton-Jones et al., 2009).

### 3.2 Parkinson’s disease (PD)

Parkinson’s disease is a neurodegenerative disorder characterized by primary symptoms of body movement slowness (bradykinesia) and another symptom, resting tremor or rigidity. Other symptoms that may occur include loss of smell and mood disturbances (Mirpour et al., 2018; Kabra et al., 2018; Alexoudi et al., 2018). Generally, the first symptom to appear due to this disease is tremor, and symptoms that arise in the late stage include postural instability, a symptom that can significantly impair the patient’s quality of life. This disease commonly occurs with advancing age, with a slow but progressive onset (Zafar and Yaddanapudi, 2023).

The basic pathophysiology of Parkinson’s disease is closely related to genetics and environmental factors that can trigger the accumulation of alpha-synuclein in the brain and loss of dopamine (Gratton et al., 2019). Additionally, Parkinson’s disease can be triggered by various interrelated etiologies including genetic susceptibility, environmental factors, mitochondrial dysfunction, oxidative stress, and protein aggregation. Furthermore, age is strongly associated with the incidence of Parkinson’s disease. The prevalence of Parkinson’s disease increases significantly with advancing age (Zafar and Yaddanapudi, 2023; Ehgoetz Martens et al., 2018; Chung et al., 2018).

In Parkinson’s disease, it is believed that all symptoms, including motor nerve disturbances in patients, are caused by one crucial area in the brain, namely, the substantia nigra. The part of the substantia nigra most affected by pathological changes is the dopaminergic neurons of the pars compacta, and this is highly evident with aging (Reeve et al., 2013). Studies indicate that 1/3 of 750 geriatrics show neuron loss in the substantia nigra ranging from mild to severe levels (Buchman et al., 2012). Furthermore, other research suggests that other parts of the brain do not undergo pathological changes compared to the substantia nigra area. Neuronal counts remain stable in geriatrics in areas such as the hippocampus, putamen, medial mammillary nucleus, hypothalamus, and the nucleus basalis of Meynert (Pakkenberg and Gundersen, 1997; Hirsch et al., 1987).

As age advances, the characteristics of neurons in the substantia nigra undergo alterations. Neurons in the substantia nigra exhibit increasing mitochondrial dysfunction with age (Bender et al., 2006; Kraytsberg et al., 2006). Additionally, dopamine within substantia nigra neurons becomes more susceptible to a decline in protective function by dopamine transporters and vulnerability to oxidative stress in aging conditions (Ma et al., 1999). Oxidative stress can also initiate calcium dysregulation in neurons in the substantia nigra. Furthermore, the increase in iron concentration in the substantia nigra with aging from the Fenton reaction also elevates the risk of developing Parkinson’s disease through mitochondrial dysfunction (Reeve et al., 2013). Moreover, the protective effect of neuromelanin against oxidative stress increases with age (Fedorow et al., 2006; Halliday et al., 2005).

The effects of mitochondrial dysfunction in aging can be examined through specific mechanisms. Respiratory deficiency in mitochondria is commonly found in aging individuals (Bender et al., 2006; Kraytsberg et al., 2006; (Itoh et al., 1996). This respiratory deficiency condition leads to a decrease in ATP production, resulting in abnormal neuronal excitability (Pissadaki and Bolam, 2013). Additionally, Parkinson’s disease is also associated with the accumulation of mitochondrial DNA deletions. In aging, mitochondrial DNA deletions are found in substantia nigra neurons compared to other brain regions. In animal model studies, it has been found that mitochondrial DNA deletions can reduce dopaminergic neurons, a key factor in the development of Parkinson’s disease (Reeve et al., 2013).

In addition to mitochondrial dysfunction, Parkinson’s disease is also triggered by factors related to impairment in the protein degradation process. In the context of aging, two main pathways involved in protein degradation, namely, the ubiquitin proteasome system (UPS) and autophagy, exhibit significant functional decline (Li and Li, 2011; Jana, 2012; Rubinsztein et al., 2011). Decreased UPS function can lead to neurodegenerative conditions and the formation of Lewy body-like inclusions (Emmanouilidou et al., 2010; Bedford et al., 2008). Conversely, reduced autophagy function can result in the accumulation of protein aggregates and mitochondrial dysfunction (Hara et al., 2006; Komatsu et al., 2006).

### 3.3 Heart failure (HF)

Heart failure is a pathological condition characterized by typical symptoms such as dyspnea, ankle swelling, and fatigue, and can be accompanied by signs such as elevated jugular venous pressure, pulmonary crackles, and peripheral edema. Heart failure is caused by both structural and functional disturbances of the heart that lead to a decrease in cardiac output and/or an increase in intracardiac pressure during rest or stress (Ponikowski et al., 2016). Generally, heart disturbances result in dysfunction of ventricular diastole and/or systole. Additionally, abnormalities of the valves, pericardium, endocardium, heart rhythm, and heart conduction may also play a role in heart failure (Schwinger, 2021).

The classification of heart failure can be determined through a combination of clinical symptoms and patient risk factors (Ponikowski et al., 2016; Tanai and Frantz, 2015). Risk factors for the occurrence of heart failure include coronary heart disease, hypertension, diabetes, family history of heart failure, obesity, chronic obstructive pulmonary disease, chronic inflammation or infection, metabolic diseases, alcohol abuse, and treatment with cardiotoxic agents. Additionally, advancing age is a risk factor for heart failure. In European and North American men aged 40, 1 in 5 has a risk of heart failure, and as age increases, the risk of heart failure also increases (Boekel et al., 2020; Cardinale et al., 2015).

The aging process significantly affects changes in the cardiac cycle during the diastolic phase. These changes include a decrease in diastolic filling rate and a reduction in the E/A ratio, accompanied by atrial enlargement (Bryg et al., 1987; Miyatake et al., 1984; Gerstenblith et al., 1977). Furthermore, aging also affects the maximal capacity of cardiac function during exercise, resulting in a decline. These changes in capacity can be observed through slowed relaxation, decreased responsiveness of beta-adrenergic receptors, and alterations in relaxation patterns (Tan et al., 2009; Spina et al., 1998; Hees et al., 2002).

In addition to influencing the cardiac cycle, aging also affects the cardiac conduction system. Changes in the conduction system may include a decrease in the level of phasic variation in the R-R interval with respiration, sinus bradycardia, and an increase in pathological sinus bradycardia (Hiss and Lamb, 1962; Fleg et al., 1990). Furthermore, there is an increase in the P-R interval, a leftward shift in the QRS axis, and a decrease in R and S wave amplitudes (Fleg et al., 1990; Golden and Golden, 1974). Additionally, atrial fibrillation, paroxysmal supraventricular tachycardia, and ventricular arrhythmia become more prevalent in aging conditions (Fleg and Kennedy, 1982; Manolio et al., 1994).

Upon closer examination, arrhythmias in the context of aging occur due to, among other factors, aberrant Ca2+ regulation. This leads to altered cytosolic Ca2+ dynamics, consequently impairing the contraction-relaxation cycle of the heart (Lakatta, 2002; Lakatta and Sollott, 2002). These changes are associated with modifications in ion currents, downregulation of SERCA2 protein levels, and increased activity of the Na+/Ca2+ exchanger (Strait and Lakatta, 2012). Additionally, alterations in lipid membrane composition due to aging exacerbate the production of reactive oxygen species (ROS), which disrupt Ca2+ regulation (Lakatta, 2002). Therefore, lifestyle interventions, particularly dietary modification with ω-3 PUFAs, show promising results in improving cardiac function in the context of aging (Strait and Lakatta, 2012).

In the cardiac adrenergic response, there is a decrease in post-synaptic β-adrenergic signaling function in aging. This results in reduced autonomic modulation of heart rate, left ventricular contractility, and arterial afterload (Lakatta, 1993a). Despite increased sympathetic neurotransmitters, there is still a decreased response to β-adrenergic antagonists in aging. This leads to an increase in plasma catecholamine concentration to counteract the decline in function and density of cardiac muscarinic β-receptors (Lakatta, 1993a; Lakatta, 1993b).

### 3.4 Atherosclerosis

Atherosclerosis is a chronic inflammatory disease affecting arterial blood vessels. It is closely associated with the accumulation of lipoproteins and active inflammation in focal areas of arterial blood vessels. Atherosclerosis can manifest in various parts of the body leading to conditions such as stroke, transient cerebral ischemia, myocardial infarction, angina pectoris, and peripheral arterial disease (Pahwa and Jialal, 2014), (Loscalzo, 2016). Atherosclerosis stands as one of the leading causes of premature cardiovascular mortality and disability (Loscalzo, 2016).

Atherosclerosis has multifactorial etiology and risk factors. Common risk factors include diabetes, hypertension, hypercholesterolemia (high LDL and cholesterol levels), smoking, male gender, lifestyle, family history of atherosclerosis, obesity, and genetics (Reiss et al., 2019; Shafi et al., 2019; Doodnauth et al., 2019). Additionally, aging is a highly dominant risk factor in the development of atherosclerosis. Aging is believed to play a significant role in the formation of atherosclerotic lesions (Head et al., 2017).

The fundamental mechanism underlying the formation of atherosclerosis is the chronic development of plaques resulting from the accumulation of lipids and chronic inflammation (Loscalzo, 2016). In the aging condition, a study conducted in mice found that immune cells derived from bone marrow progenitor cells cannot effectively repair arteries with lesions due to hyperlipidemia. This leads to arteries with lesions becoming more susceptible to plaque formation (Rauscher et al., 2003). Additionally, immune cells originating from bone marrow progenitor cells also exert a crucial protective effect on endothelial cells, namely, preventing endothelial senescence (Rauscher et al., 2003; Baker et al., 2016; Childs et al., 2016).

Additionally, in the aging condition, an abundance of protein misfolding and imperfect protein degradation has been observed (Ayyadevara et al., 2016). This state of protein misfolding and imperfect protein degradation can lead to proteotoxicity and may contribute to a range of cardiovascular-related diseases (Willis and Patterson, 2013). Furthermore, other studies have also found that upregulated protein aggregates in the aging condition are believed to be partly caused by cellular senescence (Ayyadevara et al., 2016). This could potentially represent a novel breakthrough in detecting vascular damage if upregulated protein aggregates are identified within blood vessels (Head et al., 2017).

Furthermore, in the aging condition, there is also a risk of atherosclerosis originating from hematopoietic progenitor cells. These cells may undergo mutations in their DNA, rendering them competitively advantageous in growth and leading to the formation of colonies of mutated cells (referred to as clonal hematopoiesis of intermediate potential, CHIP) (Steensma et al., 2015). One study indicates that individuals with CHIP have twice the risk of cardiovascular disease compared to those without CHIP. A gene susceptible to mutation in CHIP is Tet2, the dysfunction of which can also trigger the production of interleukin-1 beta (IL-1β), an inflammatory cytokine highly associated with atherosclerosis initiation (Jaiswal et al., 2017).

### 3.5 Type 2 diabetes (T2D)

Diabetes is a group of chronic metabolic diseases characterized by the condition of hyperglycemia. Hyperglycemia can be caused by abnormalities in insulin secretion or decreased insulin action due to insulin resistance (Halim and Halim, 2019; Zhu et al., 2021). Type 2 diabetes (T2D) constitutes 90% of diabetes cases and begins with reduced insulin sensitivity. Initially, increased insulin secretion compensates for this decline, but over time, secretion decreases, leading to uncontrolled blood sugar levels known as T2D (Goyal et al., 2024).

DM is a multifactorial condition influenced by various risk factors, all interacting to manifest the disease. In general the risk factor can be classified into non modifiable and modififiable Risk factors can generally be categorized as non-modifiable, such as ethnicity, genetic predisposition, and family history, or modifiable, including obesity, sedentary lifestyle, and high-energy-density diet (Goyal et al., 2024; Galicia-Garcia et al., 2020). Age is another significant risk factor, as the disease is strongly correlated with the aging process; older individuals have the highest prevalence of diabetes (Lee and Halter, 2017). Additionally, diabetes itself serves as a risk factor for other age-related conditions, including atherosclerosis, Parkinson’s disease, Alzheimer’s disease, stroke, non-alcoholic fatty liver disease, and cancer (Halim and Halim, 2019).

The increase in age correlates with a decrease in insulin secretion, which is known to decline by 0.7% per year with advancing age (Halim and Halim, 2019). This decline is attributed to the decreasing function of beta cells as age advances. The diminished function of beta cells is associated with a reduction in their ability to regenerate due to aging processes (Lee and Halter, 2017). The decline in the replicative capacity of cells is demonstrated by a reduction in proliferative signals, such as platelet-derived growth factor receptor (PDGFR) and PDX1. Additionally, there is an increase in cell cycle inhibitors, such as p16^INK4a^, and a prolongation of the time between cell divisions (G0) (Zhu et al., 2021; Lee and Halter, 2017).

Cellular damage accumulates as we age. DNA mutations, elevated AGEs, and IAPP accumulation are all potential forms of accumulation. DNA mutations are closely associated with ROS and are distinguished by the identification of increased transcriptional noise, genetic errors, and potential fate drift in the parent’s pancreas. 8-hydroxyguanosine levels are significantly higher in β cell DNA than in non-islet cells, suggesting that the mutational signature of β cells may be driven by guanosine oxidation. This relationship between oxidative damage and endocrine dysfunction in the pathogenesis of T2D is likely to be interpreted from this perspective. ROS can generate advanced glycation end products (AGEs) in addition to DNA mutations. These AGEs can stimulate ROS production by activating NADPH oxidase and interacting with their receptor (RAGE). IAPP is a neuroendocrine hormone that is secreted by pancreatic beta cells in conjunction with insulin. Palm amyloid, which can accumulate with age, is produced by the easy aggregation of IAPP. The incidence of IAPP deposition in patients over the age of 70 without diabetes displayed a significant increase (41.23%) in comparison to patients under the age of 70 (5.98%). Amyloid plaques can induce endoplasmic reticulum and oxidative stress which leads to β cell apoptosis (Zhu et al., 2021).

Insulin resistance is primarily influenced by body composition, particularly in those with reduced muscle mass (sarcopenia) and increased fat, particularly visceral fat (Halim and Halim, 2019). Even though insulin resistance isn't directly caused by aging, we know in general older individuals experience a decrease in physical activity resulting in obesity and loss of lean body mass (Lee and Halter, 2017).

### 3.6 Non-alcoholic fatty liver disease (NAFLD)

Non-alcoholic fatty liver disease (NAFLD) is used to describe a condition characterized by hepatic steatosis in patients without a history of chronic alcohol consumption, drug use, or hereditary diseases. The most common pathogenesis model is the “two-hit” hypothesis. The first hit involves a reduction in insulin sensitivity, which leads to the accumulation of free fatty acids in the cytoplasm of hepatocytes. The second hit pertains to injuries sustained by hepatocytes that have become more susceptible to damage. Such injuries may stem from producing reactive oxygen species (ROS), free fatty acid metabolism by cytochrome P450, fatty acid oxidation in peroxisomes, metabolism of alcohol derived from the gut, and inflammatory mediators released by adipocytes. Ultimately, hepatocytes transform and undergo ballooning, cytoskeletal aggregation, apoptosis, and necrosis (Kudaravalli et al., 2024).

The risk factors for NAFLD include obesity, diabetes, insulin resistance, dyslipidemia, and metabolic syndrome. These factors contribute to the increasing incidence of NAFLD in conjunction with the rising prevalence of these conditions (Kudaravalli et al., 2024). NAFLD is also commonly found in middle-aged and older adults, classifying it as an age-related disease (Frith et al., 2009). The impact of NAFLD in the form of hepatic and extra-hepatic complications also escalates in elderly patients (Bilson et al., 2022).

Cellular senescence plays a significant role in the pathogenesis of NAFLD. Senescence is the cell cycle cessation due to telomere shortening or the accumulation of sub-lethal DNA damage over a lifetime. In the livers of elderly individuals with NAFLD, telomere shortening is observed, a phenomenon not seen in healthy aged livers. Mitochondrial dysfunction is also believed to contribute to aging and NAFLD. Animal studies have shown that mice lacking the hepatocyte-specific PPARα gene accumulate liver fat even on a standard diet. Meanwhile, in humans with NAFLD, there is a downregulation of carnitine palmitoyltransferase 1α (Cpt1a) and acyl-CoA oxidase 1 (Acox1), leading to decreased transcriptional activity of PPARα (Li et al., 2022).

Another critical role is played by liver sinusoidal endothelial cells (LSECs), which also experience senescence with aging. The aging process results in the downregulation of vasodilators and angiocrine mediators, increased portal pressure, and loss of LSEC fenestration replaced by a basal membrane. These processes lead to increased fibrosis processes and reduced clearance of toxins (Li et al., 2022).

### 3.7 Osteoarthritis (OA)

Osteoarthritis (OA) is a chronic degenerative disease characterized by the loss of articular cartilage and remodeling of periarticular bone (Mahajan et al., 2005). OA arises from the interaction of various processes. Mechanical stress and abnormal joint mechanics, lead to the release of pro-inflammatory markers. These markers result in joint destruction through unclear mechanisms. The progression of OA begins with damage to the articular cartilage, marked by fibrillation, erosion, and irregularities. This erosion continues to advance, extending to the bone and other joint surfaces. At the cellular level, cell phenotype changes with chondrocytes becoming hypertrophic and proliferating uncontrollably. During this phase, ossification may occur, resulting in the formation of osteophytes (Sen et al., 2024).

Risk factors for OA include female gender, anatomical variations, joint injuries due to occupation or sports, muscle weakness, obesity, and age, with the latter two being the most significant risk factors (Sen et al., 2024; Loeser, 2011). It is known that up to 80% of individuals over the age of 65 have radiological manifestations of OA. However, only 60% of these individuals exhibit symptoms (Sen et al., 2024). The ageing process is associated with OA through senescence, mitochondrial dysfunction, oxidative stress, energy metabolism dysfunction, extracellular matrix communication disturbances, and age-related inflammation (Loeser et al., 2016).

As people age, there is an increase in fat mass and loss of muscle mass, leading to obesity and sarcopenia. Obesity is a well-known risk factor for OA through an increase in joint load. Other than that, fat cells are known to release adipokines and other inflammatory mediators. Obesity often accompanies muscle weakness or sarcopenia, which is also a risk factor for OA (Loeser et al., 2016). In addition to the increase in adipocyte numbers, ageing-related changes lead to increased production of IL-6 and TNF-alpha by adipocytes. The elevation of these mediators is associated with the onset and progression of OA. In elderly patients, there is a correlation between low IL-6 levels and the absence of OA (Greene and Loeser, 2015).

At the cellular level, the occurrence of senescence is one of the signs of ageing. This condition occurs in chondrocytes and is characterized by an increase in P16, which is a cell cycle inhibitor that responds to the accumulation of DNA damage (Loeser et al., 2016). Senescent cells secrete pro-inflammatory cytokines, chemokines, growth factors, and matrix metalloproteinases (MMPs). This secretion is referred to as the senescence-associated secretory phenotype (SASP), which exacerbates the progression of OA through pro-inflammatory mediators and enzymes that degrade the matrix (Greene and Loeser, 2015). In addition to senescence, there is a decline in autophagic function with age. The autophagic function shows how well a cellular recycling process is done as a protective mechanism. In chondrocytes of OA patients, there is a decrease in autophagic markers associated with increased cell death in articular cartilage (Loeser et al., 2016).

### 3.8 Osteoporosis (OP)

Osteoporosis is a chronic skeletal disease characterized by a reduction in bone mineral density and mass, leading to the deterioration of bone microarchitecture. Patients with osteoporosis face a higher risk of fractures, which overall reduces their quality of life and increases morbidity and mortality rates Osteoporosis can be categorized into primary and secondary (Porter et al., 2024). Primary osteoporosis is mainly caused by aging and sex hormone deficiency, which result in increased bone resorption and decreased bone formation (Ali, 2022). Peak bone mass is typically achieved in the third decade of life, after which it steadily declines. Secondary osteoporosis can be caused by diseases such as hyperparathyroidism, chronic kidney failure, and Cushing’s syndrome, or by medications such as corticosteroids, chemotherapy, and proton pump inhibitors. Men are more likely to develop secondary osteoporosis compared to women (Porter et al., 2024).

In the elderly, there is a decline in osteoblast function due to decreased proliferation, increased apoptosis, and senescence. Additionally, osteoprogenitor cells become dysfunctional, leading to impaired differentiation of osteoblasts. This functional decline gives rise to the accumulation of fat cells in the bone due to increased adipogenesis in the marrow. Genes such as WNT10B, RUNX2, RANKL, Osterix, Osteocalcin, OPG, and SOST have been found to exhibit abnormal expression in patients with primary osteoporosis. Markers indicating cellular senescence, such as p21, p16Ink4a, and p53, are also observed in aging bones (Chandra and Rajawat, 2021). Diminished cellular function contributes to the accumulation of microcracks due to repair failure. Microcracks increase with age, significantly impacting bone biomechanics (Sierra and Kohanski, 2016).

Beyond cellular changes, the extracellular matrix components, such as collagen and minerals, also undergo modifications during aging. In the elderly, collagen changes include non-enzymatic glycation and denaturation. Non-enzymatic glycation produces advanced glycation end products (AGEs), which can impair the mechanical function of bones. This process can occur endogenously or exogenously, such as from diet and smoking. Additionally, older adults experience increased mineralization but incomplete matrix remodeling. This process produces large, dense crystals that make bone brittle and easily fractured (Sierra and Kohanski, 2016).

Hormonal factors also play a crucial role in the pathogenesis of osteoporosis and aging. In the elderly, the function of gonadal and adrenal cells in producing estradiol, testosterone, and dehydroepiandrosterone declines. This hormonal change alters bone metabolism, leading to decreased bone mass through changes in cytokine levels. IGF-I decreases along with sex hormones, resulting in reduced bone formation. Concurrently, IL-6 increases and stimulates bone resorption (Sierra and Kohanski, 2016).

### 3.9 Chronic obstructive pulmonary disease (COPD)

Chronic Obstructive Pulmonary Disease (COPD) is a lung condition characterized by chronic respiratory symptoms, such as shortness of breath, coughing, and phlegm due to abnormalities in the airways and/or alveoli, caused by significant exposure to harmful particles or gases (Agarwal et al., 2024). Chronic inflammation causes accelerated and progressive elastic fiber damage, peribronchial fibrosis, damage to alveolar walls, microvasculature and small airways, airway remodeling, and mechanisms of chronic mucus hypersecretion. Progressive airflow limitation and airway remodeling leads to air trapping, static and dynamic hyperinflation, gas exchange abnormalities, and reduced exercise capacity and physical activity (Barnes, 2016).

In addition to smoking, which is a major risk factor in the occurrence of COPD through the reactive oxidant species produced by cigarettes, the aging process also contributes to the development and progression of COPD through various mechanisms, including the accumulation of cellular aging, mitochondrial dysfunction, and changes in intercellular communication. As aging occurs or increasing age there will also be an imbalance of inflammatory and anti-inflmmatory tissue. Lung cells of COPD patients also show SASP which is characterized by the secretion of pro-inflammatory molecules such as IL-6, IL-8, monocyte chemoattractant protein-1, and plasminogen-activated inhibitor-1, which further contributes to the pathogenesis of COPD (Easter et al., 2020).

Cellular senescence, which is a hallmark of aging, plays an important role in the pathogenesis of COPD. Senescent cells, characterized by a permanent arrest of the cell cycle, can cause chronic inflammation and tissue damage in the lungs. This is particularly relevant in COPD, where exposure to cigarette smoke causes aging of alveolar epithelial cells, leading to increased secretion of pro-inflammatory cytokines and chronic inflammation (Easter et al., 2020). Cigarette smoke can induce the expression of the cell aging marker p21 in epithelial cells and fibroblasts. Emphysematous lungs show increased expression of p16, p19 and p21, all of which are cyclin kinase inhibitors and markers of cellular aging (Chilosi et al., 2013).

As age increases, the function of stem cells and regenerative capacity decrease. This decline in stem cells contributes to the development of age-related diseases. Therefore, widespread lung inflammation results in limited stem cell proliferation capacity and successive thinning (Singer and Morrison, 2013). In addition to the mechanism of stem cell exhaustion, free radicals also contribute to aging. Mitochondrial dysfunction can lead to the release of pro-inflammatory factors and the accumulation of oxidative stress. The imbalance between pro-oxidants and antioxidants increases with age, leading to the accumulation of oxidative damage that contributes to age-related tissue injury which can exacerbate COPD symptoms (Easter et al., 2020; Spagnolo and Semenzato, 2022).

### 3.10 Benign prostatic hyperplasia (BPH)

Benign Prostatic Hyperplasia (BPH) is a disease that affects men and usually occurs in individuals aged 40 and above. One of the risk factors for the occurrence of BPH is older age, as men experience prostate growth of 2.0%–2.5% per year (Lim, 2017). The pathophysiology of BPH is caused by the enlargement of the prostate gland extending into the prostatic urethra and the bladder outlet. This enlargement of fibroadenomatous nodules leads to narrowing of the prostatic urethra, causing patients to have trouble in urinating (Lepor, 2005).

Hormonal changes occur as age increases, particularly a decrease in testosterone levels. The average serum testosterone level in plasma is around 600 ng/mL and will gradually decrease with age. Decreased testosterone levels can lead to an increase in dihydrotestosterone (DHT), which is an active form of androgen hormone in the prostate. This local production of DHT stimulates normal prostate activity but also contributes to prostate growth, which can lead to BPH. The condition where cells stop dividing due to aging can contribute to the development of BPH. Senescent cells can accumulate in prostate tissue and contribute to the development of fibroadenomatous nodules (Untergasser et al., 2005).

The occurrence of BPH is also related to metabolic syndrome including obesity, dyslipidemia, and diabetes. This metabolic syndrome leads to local inflammation and an increase in systemic adipokine and pro-inflammatory cytokine levels, including adiponectin, leptin, tumor necrosis factor (TNF), IL-6, and CCL2. This pro-inflammatory condition also increases the risk of viral and bacterial infections, which can act as stimulating factors to injure prostate cells, thus causing chronic inflammation and involved in the process of BPH proliferation. Direct involvements of bacteria and virus as infection, include; *Staphylococcus, Acinetobacter, Candida, and Trichomonas spp* have been reported (Hata et al., 2023).

The aging factor is also associated with the accumulation of oxidative stress in the body. Oxidative stress plays a role in the proliferation of BPH (Benign Prostatic Hyperplasia) associated with chronic inflammation in the prostate (Hata et al., 2023). In a study using a transgenic mouse model with specific expression of prostate NADPH oxidase 4 (Nox4) that drives the formation of nitric oxide synthase (NOS), it was shown that mice expressing Nox4 experienced increased oxidative DNA damage in the prostate, increased prostate weight, and histological changes including epithelial proliferation and fibrosis compared to wild-type mice. This indicates that oxidative stress reactions are associated with changes in epithelium and stroma (Vital et al., 2016).

### 3.11 Aging-related macular degeneration (AMD)

Age-related macular degeneration (AMD) is one of the degenerative diseases that causes progressive vision loss (Heesterbeek et al., 2020). The macula is a part of the retina filled with cone cells that are sensitive to color and responsible for high acuity vision. Macular degeneration is the most common cause and accounts for 8.7% of blindness cases worldwide. The risk of developing macular degeneration increases by 2% in individuals aged 50–59 and rises to 30% in those aged over 75 years (Wong et al., 2014).

In AMD, there is dysfunction of the retinal pigment epithelial (RPE) and photoreceptors simultaneously with the accumulation of waste material in the subretinal space (RPD) and between the RPE and choroid (drusen). With age, there is a normal decrease in RPE cell density. Some RPE cells become multinucleated, especially in the periphery, indicating cell fusion or failure of cell division, resulting in vision impairment (Wong et al., 2022). The waste deposition that occurs in AMD is often referred to as lipofuscin and melanolipofuscin. The accumulation of this waste contributes to oxidative stress on the RPE and choriocapillaris, leading to inflammation and further damage (Olchawa et al., 2017).

The aging factor also causes abnormal Extracellular Matrix (ECM) production because most of it comes from RPE and photoreceptor cells, but some also originate from cells in the choroid and substances in systemic circulation. Abnormal ECM influences further damage to the retina, RPE, and choroid because nutrient diffusion becomes impaired. Nutrient diffusion and waste product diffusion are also facilitated by Bruch’s membrane (BrM). In RPE, there is a complex of matrix proteins called Bruch’s membrane (BrM). BrM thickness increases linearly with age. Changes in thickness are also associated with changes in protein composition, protein cross-linking, increased glycosaminoglycan size, and increased lipid content, thus disrupting the diffusion of small and large molecules across BrM (Zarbin, 2004).

### 3.12 Cancer

Cancer is a condition where body cells grow uncontrollably, disregarding signals that normally prompt them to stop dividing or die, and infiltrate and destroy surrounding tissues. It arises when genetic mutations disrupt the mechanisms that regulate normal cell growth. The pathophysiology of cancer involves a series of complex events encompassing various factors, including genetics, environment, and lifestyle (Brown et al., 2023). The process of cancer metastasis, leading to advanced stages, involves the initiation, promotion, and progression of metastasis. The initiation stage begins when cancer cells detach from their primary tumor site, facilitated by epithelial-to-mesenchymal transition (EMT). Subsequently, in the promotion stage, cancer cells travel through the bloodstream to reach distant organs. This process is facilitated by specific receptors on the cell surface such as CD 36, which binds to palmitate acid to promote metastasis. The progression stage of metastasis involves the formation of secondary tumors at distant sites influenced by genetic and epigenetic modifications (Fares et al., 2020; Neophytou et al., 2021).

The pathophysiology of cancer in relation to aging is marked by a complex interaction of various factors contributing to the development of the disease. Aging is associated with a decline in gene repair, and the expression of the DNA polymerase δ1 enzyme, crucial for DNA repair, significantly decreases with age. Double-strand DNA breaks play a role in the loss of heterozygosity in the elderly and may contribute to the occurrence of cancer (Liu et al., 2016). Throughout life, the human body is exposed to numerous exogenous stimuli that contribute to mutagenic events. One hazardous exogenous exposure is free radicals, as they can trigger DNA mutations. Unrepaired DNA errors can lead to the accumulation of oncogenic traits and the development of diseases, including cancer (Smetana et al., 2016). A study showed that during aging, 3,000 to 13,000 genes per genome may be affected by 5,000 to 50,000 mutations (Bavarva et al., 2014). This is further supported by the observation that the most common occurrence of cancer sharply increases after the age of 50 due to the accumulation of oncogenic mutations within a clone (Laconi et al., 2020).

On the other hand, cellular aging leads to the cessation of the removal of senescent cells (apoptosis), without direct cell death. This is still highly effective as an anticancer barrier because it prevents the expansion of altered clonal cells. Meanwhile, longer-lasting “chronic” senescent cells can trigger carcinogenesis and tissue dysfunction (Childs et al., 2014; Ritschka et al., 2017). Aged cells express an aging-associated secretory phenotype that includes pro-inflammatory cytokines and chronic exposure. The pro-inflammatory status in aging can provide “nourishment” for cancer cells, thus contributing to the increased incidence of cancer in older individuals (Laconi et al., 2020; Bürkle et al., 2007).

**FIGURE 3:**
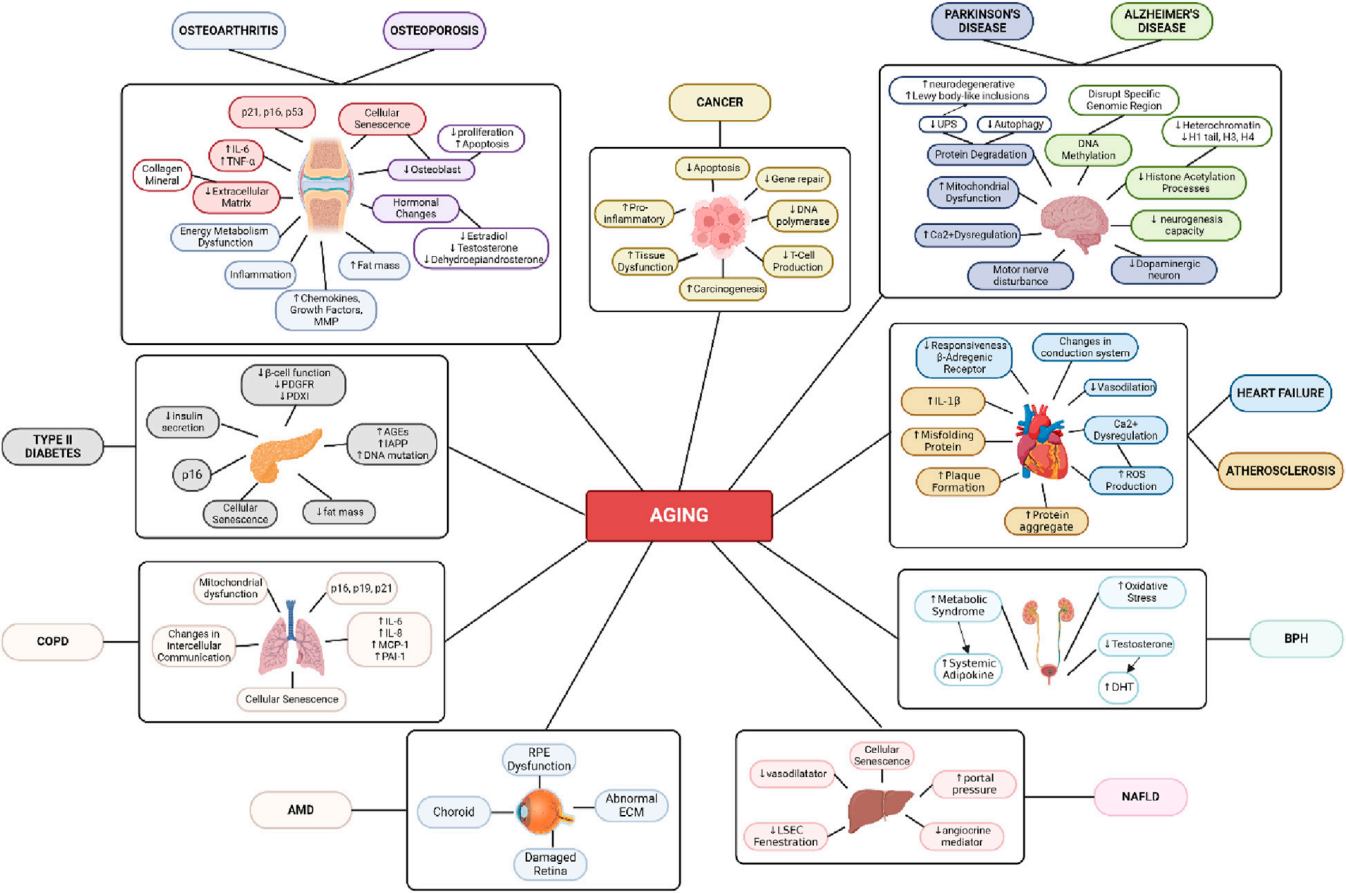
Age-Related Disease Mechanism. Created with BioRender.com Premium License by Fahrul Nurkolis (https://app.biorender.com, accessed on 10th July 2024).

The immune system also becomes less effective as age increases. The thymus, which is a vital organ in the development and maturation of T cells, shrinks from around the age of 1 year and continues to diminish. In elderly individuals, the thymus is replaced by fatty tissue. The decline in thymus function leads to a decrease in T cell production, resulting in the body being less effective at detecting and eliminating cancer cells as age advances (Figure 3). Weakening of the immune system can also increase the risk of cancer by allowing cancer cells to grow and persist in the body (Foster et al., 2011).

## 4 Oral microbiome and aging-related diseases

### 4.1 Alzheimer’s disease

It is well known that Alzheimer’s disease correlates with changes in oral microbiome that are linked to the persistence of illness. Due to the effects of Alzheimer’s disease, many people’s motor skills are impaired, such as their ability to perform daily tasks and maintain their oral health. This makes microbioma more effective in treating periodontitis and dental cavities (Greene and Vermillion, 1960). In addition, research conducted in 2013 revealed a connection between Alzheimer’s disease and a patient’s quality of life that is influenced by dental health issues (Cicciù et al., 2013). After that, research conducted on 59 participants by de Souza Rolim (2014) revealed that patients with Alzheimer’s disease were more likely to experience orofacial pain and periodontitis (de Souza Rolim et al., 2014).

Dental caries is one of the most common infections in children and has a strong correlation with socioeconomic status, lifestyle, genetics, and oral microbiome conditions (Takahashi and Nyvad, 2011). After a few years, it was discovered that Alzheimer’s disease had a strong correlation with dental caries. Research conducted by Jones (1993) shown that the early characteristics of patients with dental caries were significantly higher in those with moderate to advanced Alzheimer’s disease (Jones et al., 1993). In addition, the study by Ellefsen (2012) also demonstrated that people with Alzheimer’s disease had a higher number of coronal and root caries than people with dementia or people without dementia (Ellefsen et al., 2012).

Periodontitis is a type of oral infection caused by anaerobik gram-negative bacteria that can form biofilms in subgingival tissues and cause localized or systemic infections by causing various inflammatory mediators (Sureda et al., 2020). The first link between peridontitis and Alzheimer’s disease is inherited variation in the IL-1 gene family, which is associated with various inflammatory responses and the progression of chronic diseases, the most severe of which is Alzheimer’s disease (Kornman, 2006). Research conducted by Kubota et al. (2014) indicates that the components of the Alzheimer’s disease pathway significantly increase in patients with periodontitis who experience gingival gingival inflammation (Kubota et al., 2014). Other studies have also confirmed the association between periodontitis and Alzheimer’s disease relasi, namely, by measuring tumor necrosis factor alpha (TNF-α) levels, which are higher in patients with Alzheimer’s disease with periodontitis compared to those with the condition without periodontitis (Farhad et al., 2014). Table 1 shows the oral microbiota that is typically present in Alzheimer’s disease progression.

**TABLE 1:** Oral microbiota related to development of Alzheimer’s disease.

No.	Studies	Outcomes	References
1	This is a cross sectional study that assess the relationship between periodontitis and cognitive impairment in older adult	• Individuals with high levels of Porphyromonas gingivalis, a periodontitis pathogen, have significantly greater odds of experiencing impairments in verbal memory and subtraction test performance	Noble et al. (2009)
2	This study investigates oral *Treponema* population in human brain and the association with alzheimer’s disease	• Alzheimer’s disease patients’ ganglia had *treponema*, and PCR could detect six out of seven types of the disease • Monoclonal antibodies against *Treponema pectinovorum* and *Treponema socranskii* were detected within the trigeminal ganglia’s badan neuron	Riviere et al. (2002)
3	This study investigates if lipopolysaccharides induce patomechanism of tau pathology	• Lipopolysaccharides from *Salmonella* abortus can affect pathologies associated with Alzheimer’s disease	Lee et al. (2010)
4	This study assesses serum antibody of bacteria in oral peridontal disease of participant converted to AD and control	• There was a significant increase in antibodies to *Prevotella intermedia* and *Fusobacterium nucleatum* in participants of alzheimer’s disease and mild cognitive impairment • Then, there was a significant increase in the antibodies of *Treponema denticola* and Porphyromonas gingivalis in Alzheimer’s disease	Stein et al. (2012)
5	This study examines the relation between alzheimer’s disease and herpes simplex virus-1 (HSV-1) and to understand the effect of HSV-1 on alzheimer’s disease progression	• Studies of proteins that interact with HSV-1 in connection with their presence in plaque and amyloid fibrillation in Alzheimer’s disease showed a very significant increase in the known binding proteins of HSV-1, in these structures	Carter (2011)

### 4.2 Parkinson’s disease

The correlation between oral microbiota and Parkinson’s disease has been identified. Poor oral health is highly prevalent among Parkinson’s disease patients (Hanaoka and Kashihara, 2009). Table 2 demonstrates changes in oral microbiota associated with Parkinson’s disease.

**TABLE 2:** The heightened and lowered oral microbiota in Parkinson’s disease.

No.	Studies	Outcomes	References
1	This study investigates if there is a link between oral and nasal microbiota with parkinson’s disease	• Parkinson’s disease patients exhibited a higher prevalence of the Prevotellaceae family compared to the control group • Additionally, there was an elevated occurrence of the Prevotella genus in association with a decline in oral hygiene • Furthermore, Parkinson’s disease patients displayed an increased presence of Moryella and Erysipelotrichaceae	Pereira et al. (2017)
2	This study examines which biomarker that is sensitive and specific in change of oral microbiome in parkinson’s disease	• Parkinson’s disease patients show an elevated presence of Bifidobacteriaceae and Lactobacillaceae • The Enterobacteriaceae family is reduced and there are alterations in the abundance of the *Bacillus* genus in Parkinson’s disease patients • Parkinson’s disease is associated with an increased occurrence of the Saccharomycetaceae family, including *Candida* and *Saccharomyces cerevisiae* • The Acidaminococcaceae family is elevated and there are changes in the abundance of the Flavobacteriaceae family in Parkinson’s disease • The early stage of Parkinson’s disease is characterized by an increase in the Rhodococcus genus	Mihaila et al. (2019)
3	This study explores the composition of oral microbiota and level of oral inflammation in parkinson’s disease patients	• Parkinson’s disease patients exhibit elevated levels of *Streptococcus* mutans, Veillonella, Lactobacillaceae, Scardovia, Kingella oralis, Negativicutes, Prevotella, and Firmicutes in their oral microbiome • The Prevotellaceae family (specifically, Aloprevotella AM420222 s, Alloprevotella PAC001345 s, Prevotella PAC001346 s, and Prevotella histicola) experienced an increase in bacteria • Parkinson’s disease patients showed a drop in the SR1 phylum	Fleury et al. (2021)
4	This study analyses the impact of poor oral health, poor oral hygiene, and dysphagia status on the oral microbiota composition of parkinson’s disease patients	• There was an increase in *Streptococcus* pneumoniae and *Lactobacillus* sp. in Parkinson’s disease patients	Rozas et al. (2021)

One of the oral bacteria, *Streptococcus mutans*, has the ability to form an amyloid protein, a protein associated with the pathophysiology of Parkinson’s disease. In models of mice exposed to oral bacteria producing amyloid proteins, there was increased aggregation and production of alpha-synuclein and cerebral inflammation (Fleury et al., 2021). Besides, oral bacteria Prevotella are opportunistic bacteria. In PD disease, an increase in Prevotella is suspected to be associated with motor and non-motor weaknesses inining oral care (Pereira et al., 2017).

### 4.3 Diabetes

In diagnosing diabetes, diversity changes cannot be a reliable biomarker for diabetes. From 4 studies, only two observed diversity changes in the diabetes population (Saeb et al., 2019; Almeida-Santos et al., 2021; Lee et al., 2022). However, a decrease in diversity is still an indicator of diabetes due to dysbiosis caused by a decrease in the relative presence of non-dominant genera or an increase in pathogenic bacteria.

*Veilonella genera* abundance was higher in T2D patients in 2 studies (Table 3). In patients with diabetes, a shift in the metabolism leading to an increase in lactate leads to acidification of the oral environment. As a response to protect the oral microbiome, *Veilonella* and other acidogenic bacteria like *Prevotella* and *Leptotrichia* number are increasing because of their ability to further metabolite lactate (Agarwal et al., 2024; Shaalan et al., 2022). Acidification concurrent with immune dysfunction also leads to an increase of pathogenic and a decrease of probiotic bacteria in the oral cavity of diabetes patients (Saeb et al., 2019).

**TABLE 3:** Clinical studies of oral microbiota related to T2D.

No	Studies	Outcomes	References
1	This is a case-controlled study comparing the salivary microbiome of people with and without T2D and its relation to obesity status	• Patients with T2D have a decreased alpha diversity compared to the non-T2D • The *Veillonella and Lactobacillus* genera abundance increases in T2D patients • The *Tannerella* and *Dialister* genera decrease in T2D patients • Saliva type 1, which has a lower diversity and higher proportion of *Streptococcus*, Rothia, and Veillonella, was more common in T2D patients • Saliva type 3, which has a higher proportion of *Neisseria* and *Porphyromonas,* was more common in non-T2D patients	Shaalan et al. (2022)
2	A cross-sectional and case-controlled study comparing the oral microbiome characteristics in normoglycemic, pre-diabetes, and type 2 diabetes	- Microbiome diversity is reduced in patients with prediabetes and diabetes compared to non-diabetes patients. Diabetes patients have the lowest diversity of microbiome - The decrease in diversity in diabetic patients is attributed to an increase in pathogenic species. In diabetic patients, 38.5% of the bacteria are pathogenic, and no probiotic bacteria are present - Pathogenic bacteria identified in pre-diabetic and T2D groups include *Staphylococcus warneri, Leptothrix* sp.*,* and *Streptococcus downei*	Saeb et al. (2019)
3	The analysis of saliva microbiomes in patients with or without diabetes undergoing uncovering procedures following implant placement	- No significant difference in alpha diversity was found between the two groups - Significant differences were only observed in the family Corynebacteriaceae and the genus Corynebacterium, both of which were more abundant in the non-diabetic group	Lee et al. (2022)
4	A cross-sectional observational study was conducted to review the composition of saliva microbiomes in diabetic patients and healthy volunteers	- Bacteroidetes, Proteobacteria, and Firmicutes are the most abundant phyla in both the diabetes and non-diabetes groups - Saliva from diabetic patients contains more Bacteroidetes and fewer Proteobacteria compared to non-diabetic individuals - There is a significant increase in the genera Prevotella, Porphyromonas, Veillonella, Leptotrichia, *Lactobacillus*, and *Streptococcus* in the diabetes group compared to the non-diabetes group - Genera *Neisseria* and Capnocytophaga are significantly higher in the non-diabetes group compared to diabetes	Agarwal et al. (2024)
5	Comparing salivary microbiome of 25 T2D patiens to 25 healthy volunteers	- There’s no difference in alpha and beta diversity between T2D and healthy gorup - Betaproteobacteria were significantly higher in T2D group - Deltaproteobacteria, Spirochaetes, Mollicutes, and Synergistia were significantly higher in healthy group	Almeida-Santos et al. (2021)

### 4.4 NAFLD

In NAFLD patients, Wang’s study found an increase in alpha diversity. This result contradicts the general understanding that high diversity is a marker of a healthy ecosystem. However, this increase in diversity aligns with previous research on the same disease group, examining the microbiome in supragingival plaque. These findings suggest that the increase in diversity results from poor oral health due to plaque accumulation and gum bleeding becoming a new nutrient source. NAFLD is known to alter the composition of the salivary microbiome, increasing the risk of oral diseases and poor oral health (Wang et al., 2023).

As found in diabetes, a decrease in Proteobacteria was observed in the MAFLD group. The reduction of Proteobacteria in MAFLD could be due to their role in maintaining glucose homeostasis and reducing inflammatory responses. Therefore, a decrease in Proteobacteria is commonly seen in metabolic disorders like diabetes and MAFLD (Agarwal et al., 2024; Wang et al., 2023).

In addition, an increase in Firmicutes was observed in the MAFLD group. Unlike Proteobacteria, Firmicutes are involved in the fermentation and metabolism of carbohydrates and lipids, increasing their presence in metabolic disorders. The interaction between these two bacteria is often expressed as the Firmicutes/Proteobacteria ratio. An increased ratio, indicating a competitive advantage for Firmicutes relative to Proteobacteria, is found in various diseases characterized by low-grade chronic inflammation, such as MAFLD, schizophrenia, and Sjogren’s syndrome (Wang et al., 2023).

Wang’s study also identified *Fretibacterium*, *Neisseria, Treponema, Delftia, Capnocytophaga, Dialister,* and *Erysipelotrichaceae_UCG-003* as seven genera that can serve as biomarkers with an AUC of 0.82 (Table 4), indicating their clinical usefulness in diagnosing MAFLD (Wang et al., 2023; Çorbacıoğlu and Aksel, 2023). Niu’s research reviewed the use of fungi as biomarkers and found Mucor ambiguus to be significantly different between diseased and healthy individuals. However, its clinical utility is limited, with an AUC of 0.76 below the threshold of 0.80 (Niu et al., 2023).

**TABLE 4:** Oral microbiota related to NAFLD.

No	Studies	Outcomes	References
1	Reviewing the saliva microbiome in 10 NAFLD/MAFLD patients and 10 healthy patients using 16S rRNA amplicon sequencing and bioinformatics analysis	- There was an increase in the relative abundance of Firmicutes and a decrease in Proteobacteria in the NAFLD group compared to the healthy group - A panel detecting 7 genera, namely, Fretibacterium, *Neisseria*, *Treponema*, Delftia, Capnocytophaga, Dialister, and Erysipelotrichaceae_UCG-003, was identified as an optimal biomarker with an AUC of 0.82 - Alpha diversity, measured by the Ace and Chao1 richness indices, was significantly higher in the MAFLD group compared to the NAFLD group	Wang et al. (2023)
2	Reviewing the fungal microbiome in saliva, feces, and supragingival plaque in 21 NAFLD/MAFLD patients and 20 healthy patients using 16 S rRNA amplicon sequencing and bioinformatics analysis	- Mucor ambiguus increased in saliva samples of MAFLD patients compared to controls	Niu et al. (2023)

### 4.5 Osteoarthritis

Only one study has examined microbiome changes in patients with osteoarthritis. This study found an increase in alpha diversity, which contrasts with other chronic inflammatory diseases (Chen et al., 2018) (Table 5). A decrease in Proteobacteria and an increase in Firmicutes led to a higher Firmicutes/Proteobacteria ratio, observed in OA, consistent with other conditions such as MAFLD and diabetes (Wang et al., 2023; Chen et al., 2018). The study also identified eight OTUs—Actinomyces, *Neisseria*, *Neisseria* subflava, *Haemophilus* parainfluenzae, *Haemophilus*, Veillonella dispar, and Prevotella—as the most accurate biomarkers with an AUC of 0.87, indicating clinical usefulness (Chen et al., 2018).

**TABLE 5:** Oral microbiota related to development of osteoarthritis.

No	Studies	Outcomes	References
1	Reviewing the salivary microbiome in 155 healthy controls, 110 rheumatoid arthritis (RA) patients, and 67 osteoarthritis (OA) patients. The ages of the subjects were 49.96 ± 11.17, 56.65 ± 11.36, and 57.79 ± 9.712, respectively	5 Eight OTUs—Actinomyces, *Neisseria*, *Neisseria* subflava, *Haemophilus* parainfluenzae, *Haemophilus*, Veillonella dispar, Prevotella, and Veillonella—are identified as potential biomarkers with the highest AUC of 0.87 6 Healthy patients have a higher relative abundance of Proteobacteria compared to patients with rheumatoid arthritis (RA) or osteoarthritis (OA) 7 Healthy patients have a lower relative abundance of Firmicutes compared to patients with RA or OA. 8 Alpha diversity is increased in OA patients compared to the healthy group	Chen et al. (2018)

### 4.6 COPD

The correlation between oral microbiota and COPD has been identified. Table 6 demonstrates changes in oral microbiota associated with COPD. According to Wu’s (2017) research, it was found that COPD patients had higher levels of oral bacteria from the genera Dysgonomonas, Desulfobulbus, and Catonella, as well as *P. intermedia, P. endodontalis, D. wimpennyi,* and *C. morbi*. These bacteria are believed to contribute to the development of COPD. In addition to an increase in the overall number of oral bacteria, the study by Wu (2017) also observed a decrease in the specific genera of oral bacteria, namely, *Oribacterium*, *Streptomyces*, and *Arcanobacterium* (Wu et al., 2017). In Lin’s (2020) study, there was a notable rise in the bacteria genera *Rothia, Veillonella, and Actinomyces*, along with a large decline in the genus *Fusobacterium*. This suggests that there is a correlation between COPD exacerbating the symptoms of periodontitis, or the management of periodontitis exacerbating COPD (Lin et al., 2020).

**TABLE 6:** The heightened and lowered oral microbiota in COPD.

No	Studies	Outcomes	References
1	Characterization of periodontal microbiome from 55 COPD (n = 30 patient COPD with periodontitis (age 65.2 ± 7.4) and n- 25 patient COPD without periodontitis (age 65.6 ± 7.1) and 50 non-COPD (n = 25 patient with periodontitis (age 63.4 ± 7.0)) and n = 25 patient without periodontitis (age 64.8 ± 6.7) using 16S rRNA analysis gene metagenomic sequencing	- Patients with COPD exhibit a decrease in both the richness and diversity of bacteria in the periodontal tissues of individuals without periodontal disease - Three genera (*Johnsonella*, *Campylobacter*, and *Oribacterium*) and five species (J. *ignava*, P. *canis*, *Fusobacterium simiae, Campylobacter showae*, and *Gemella cunicola*) were found to be associated with COPD but not with periodontitis - The genera *Johnsonella* and J. *ignava* were linked to COPD but not to periodontitis. These genera are associated with oral squamous cell carcinoma - In this study, six genera related to periodontitis and eight species were identified in COPD patients. Genera *Dysgonomonas, Desulfobulbus*, and *Catonella*, as well as *P. intermedia, P. endodontalis, D. wimpennyi, and C. morbi*, were more abundant in COPD patients compared to non-COPD patients. Increasing of these microorganisms in periodontal tissue could be related to the development of COPD. - P. *intermedia* induces strong expression of antimicrobial peptides and IL-8, leading to migration of innate immune cells to the local infection site, resulting in an imbalance in local flora and inflammation - A decrease in the genera *Oribacterium, Streptomyces, and Arcanobacterium* was observed in the periodontal tissues of COPD patients. Understanding the microbial relationships and differences between COPD and periodontitis may provide a new strategy for the diagnosis, monitoring, and treatment of COPD.	Wu et al. (2017)
2	Investigated saliva microbiome of patients with COPD and periodontitis (n = 21 (age 60.36 ± 8.78)) compared with patients periodontitis (n = 36 (age 59.75 ± 8.00)), and healthy controls (n = 14 (age 60.24 ± 8.84)) using pyrosequencing of polymerase chain reaction-amplified 16s rRNA genes	- Indicates that the bacterial genera *Rothia, Veillonella*, and *Actinomyces* significantly increase during periodontitis and increase even more in patients with both periodontitis and COPD compared to healthy individuals - The bacterial genus *Fusobacterium* significantly decreases during periodontitis and decreases even more in patients with both periodontitis and COPD compared to healthy individuals	Lin et al. (2020)
3	Characterization oral microbiome from subgingival plaque and gingival crevicular fluid samples from healthy controls (n = 31 (age 25 (23–38)), patients with periodontitis (n = 24 (age 53.5 (47.25–61.25)), patients with COPD (n = 28 (age 61 (51,75–67.75)), and patients with both periodontitis and COPD (n = 29 (age 66 (60.5–72.5)) using16S rRNA gene sequencing	- According to LEfSe analysis in the GCF samples, the genera Mogibacterium increased in the group with COPD without periodontitis, while in patients with periodontitis and COPD, there was an increase in the genera *Phocaeicola* and *Schwartzia* - According to DESeq2 analysis, the genera *Serratia* decreased in COPD patients, while in patients with periodontitis and COPD, the genera *Alloprevotella, Kingella,* and *Dialister* experienced a decrease	Liu et al. (2023)

### 4.7 BPH

Periodontitis has been linked to various systemic diseases. The potential connection between periodontitis and systemic ailments might involve factors like metastatic infections, the spread of bacterial toxins, and immune dysfunction. Oral bacteria have the capability to directly invade endothelial cells independent of their byproducts. In this study by Estemalik et al. (2017) mentioned plausible that these oral pathogens travel via the bloodstream to reach the prostate gland and infiltrate its epithelial cells (Table 7). Notably, *Porphyromonas gingivalis* (Pg) has been shown to penetrate gingival epithelial tissue and was observed more frequently in prostatic secretions, suggesting a need for further validation in forthcoming investigations. Moreover, Pg in the oral cavity releases Arg-gingipain, contributing to collagen degradation, while *Treponema denticola* (Td) secretes chymotrypsin-like proteinase, enhancing epithelial permeability and initiating periodontal tissue breakdown. These organisms may exert a similar effect upon reaching the prostate, penetrating intraepithelial tissue and inducing histological alterations in the organ. The study found evidence of similar bacterial DNA in both prostatic secretion and subgingival dental plaque from the same individuals, indicating a potential link between oral pathogens and prostatic inflammation (Estemalik et al., 2017).

**TABLE 7:** Oral microbiota related to development of BPH.

No	Studies	Outcomes	References
1	Investigated oral pathogens from expressed prostatic secretions of patients with periodontal disease and chronic prostatitis (n = 14) or benign prostatic hyperplasia (n = 10) (age 61.2 ± 14.3) using PCR Amplification	- In total 17 patients was evaluated on periodontal examination and plaque sample, *E. coli* was present in 52.9% (9/17) in all dental plaque samples. *E. coli* is a pathogen most commonly associated with prostatitis and urinary tract infections - In prostatic sample, *Treponemua denticola* was present in 47.4% (9/19), *Porphyromonas gingivalis* in 45.8% (11/24), *E. Coli* in 36.8% (7/19), and *Prevotella intermedia* was not detected in any sample - Fifteen patients had both prostatic secretion and plaque samples tested for *Porphyromonas gingivalis*, *Treponema denticola*, *Prevotella intermedia*, and *E. coli* - A man with prostate inflammation will have a higher prevalence of oral pathogens within the glands - Pathogen from a distant site like periodontium play role in sustaining a low-grade prostatic inflammatory process until continue progress to BPH	Estemalik et al. (2017)

### 4.8 Aging related macular degeneration

The correlation between oral microbiota and aging related macular degeneration has been identified. Table 8 demonstrates changes in oral microbiota associated with aging related macular degeneration. Rullo’s (2020) research indicates that the *Actinobacteria* phylum contains prevalent oral bacteria. Furthermore, the presence of oral microbial pathogens such as *Rothia, Proprionbacteriaceae,* and *Corynebacteriaceae* has been linked to the development of aging-related macular degeneration. This association is attributed to their ability to promote inflammation, as explained by Rullo in 2020 (Rullo et al., 2020).

**TABLE 8:** The heightened and lowered oral microbiota in aging related macular degeneration.

No	Studies	Outcomes	References
1	Investigated local oral and nasal microbiome in newly diagnosed neovascular age-related macular degeneration (n = 13) compared with controls (n = 5) using PCR amplification of 16S rRNA genes with universal primers amplifying the V4 variable region (515f-806r)	- In all biospecimen of swab buccal (oral mucosa) in patients with newly diagnosed AMD compared to controls, showed dominant phylum with members of *Actinobacteria* being the greatest - The top three of oral microbiome identified as dfifferent compared with control sample included *Rothia* (genus), *Proprionbacteriaceae* (family), *Corynebacteriaceae* (genus) - Microbial pathogens found in the oral cavity such as *Rothia, Proprionbacteriaceae, Corynebacteriaceae* are typical in newly diagnosed neovascular AMD patients. These bacteria may represent pathogens associated with increased inflammation linked to the pathogenesis of AMD partly regulated by the innate immune system. There is a possibility that local changes in microbial composition drive increased regulation of pro-inflammatory pathways in distant organs including the choroid/RPE	Rullo et al. (2020)

### 4.9 Cancer

In the study including below the tabel, mentioned that oral microbiome has been linked various cancer such as oral esophageal squamos cell carcinoma, pancreatic cancer, liver carcinoma, lung cancer, oral squamos cell carcinoma, colorectal cancer, digestive tract cancer, gastric cancer Tongue coating microbiome data (Farrell et al., 2012; Chen et al., 2015; Torres, 2015; Yan et al., 2015; Kato et al., 2016; Lu et al., 2016; Lu et al., 2019; Olson et al., 2017; Peters et al., 2017; Fan et al., 2018; Flemer et al., 2018; Sun et al., 2018; Yang et al., 2018; Kageyama et al., 2019; Zhang et al., 2019; Vogtmann et al., 2020). The oral microbiome undergoes changes with age, leading to dysbiosis, which is characterized by an imbalance of the normal microbial flora.

In oral esophageal cancer include the study by Peters et al. (2017) the abundance of periodontal pathogen *Porphyromonas gingivalis* trended higher risk with mechanism the presence of *Porphyromonas gingivalis* was associated with ESCC lymph node metastasis and decreased survival time (Peters et al., 2017). Increase *Prevotella* associated with oral hygeine, in individuals with poor oral hygeinge tend to develop a more varied and intricate bacterial community, primarily composed of anerobic gram-negative bacteria such as *Prevotella* (Chen et al., 2015).

In pancreatic cancer include study by Fan et al. (2018) found that *P. gingivalis* and *A. actinomycetemcomitans* are key bacterial agents in the development of periodontal disease and subsequent tooth loss. Studies have indicated a link between a history of periodontal disease and tooth loss and an increased risk of pancreatic cancer. P. gingivalis has been observed to potentially evade the immune system by infiltrating host cells and disrupting signaling pathways, including cytokine and receptor degradation. Furthermore, both P. gingivalis and A. actinomycetemcomitans can trigger Toll-like receptor (TLR) signaling pathways, which are known to play a crucial role in pancreatic carcinogenesis in animal models (Fan et al., 2018). In study by Farrel et al. (2012) in patient with pancreatic cancer can elevated *G. adiacens* typically regarded as opportunistic pathogens, might have links to systemic inflammations (Farrell et al., 2012). In study by Torres (2015) increase level in *Leptorichia and Pophyromonas. Leptorichia* have been detected in the bloodstream of immunocompromised individuals. *Leptotrichia* has also been recovered from cardiovascular and gastrointestinal abscesses, systemic infections, indicating their potential pathogenicity. Regarding Porphyromonas, the presence of antibodies to Porphyromonas gingivalis has been directly linked to pancreatic cancer (Torres, 2015; Vogtmann et al., 2020). In patient with pancreatic adenocarcinoma found that the increase level of *Enterobacteriaceae, Lachnospiraceae G7, Bacteroidaceae* or *Staphylococcaceae* (Vogtmann et al., 2020). The elevated level of *Bacilli, Lactobacillales, Streptococcaceae, Streptococcus, Streptococcus thermophilus* found in population with pacreatic ductal adenocarcinoma (Olson et al., 2017). Increase level of *Leptotrichia, Fusobacterium, Rothia, Actinomyces, Corynebacterium, Atopobium, Peptostreptococcus, Catonella, Oribacterium, Filifactor, Campylobacter, Moraxella* and *Tannerella* bacteria found in patient with pacreatic head carcinoma (Lu et al., 2019).

In liver carcinoma study by Lu et al. (2016) shown that *Fusobacterium nucleatum* promotes liver metastasis in colorectal cancer by modulating the liver immune microenvironment. It increases the levels of pro-inflammatory cytokines such as IL6, IL12, IL9, IL17A, CXCL1, MCP-1, TNF-α, and IFN-γ in the plasma, leading to a pro-inflammatory state in the liver (Yin et al., 2022). *Oribacterium* is significantly more abundant in liver tumor tissues compared to para-cancerous tissues, indicating its potential role in liver carcinoma (Liu et al., 2023).

In this study found bacteria in the lung cancer devided into two type including squamos cell carcinoma lung cancer shown the increase *Capnocytophaga* and *Veillonella* bacteria and in study with patient non-small cell lung cancer shown that increase in *Firmicutes* and two genera *Streptococcus* and *Veillonella. Capnocytophaga* can contribute to the development of lung cancer by altering the immune response and promoting chronic inflammation (Yan et al., 2015; Zhang et al., 2019).

In oral squamous cell carcinoma found that the increase in *Fusobacterium periodonticum, Parvimonas micra, Streptococcus constellatus, Haemophilus influenza, and Filifactor alocis* (Yang et al., 2018). *Fusobacterium periodonticum* infection increases PD-L1 expression in head and neck SCC cell lines, which may contribute to immune evasion and tumor propagation. *Haemophilus influenza* can contribute to the development of oral cancer by altering the immune response and promoting chronic inflammation (Michikawa et al., 2022).

In colorectal cancer found that increase *Lactobacillus, Rothia, Streptococcus, Prevotella* (Kato et al., 2016; Flemer et al., 2018). Lactobacilli, commonly found in the mouth, are strongly linked to tooth decay. While it's unlikely that *Lactobacilli* directly cause cancer in the digestive system, their presence may indicate poor oral hygiene or health. Studies show connections between periodontal disease and increased risk of mortality, colorectal cancer, and positive results in colorectal cancer screening. *Rothia*, typically considered harmless in the mouth, has been identified as an opportunistic pathogen causing various illnesses in those with weakened immune systems, including periodontitis in AIDS patients. Even though our cases didn't receive chemotherapy, individuals with or recovering from cancer might experience compromised immune function (Kato et al., 2016).

**FIGURE 4:**
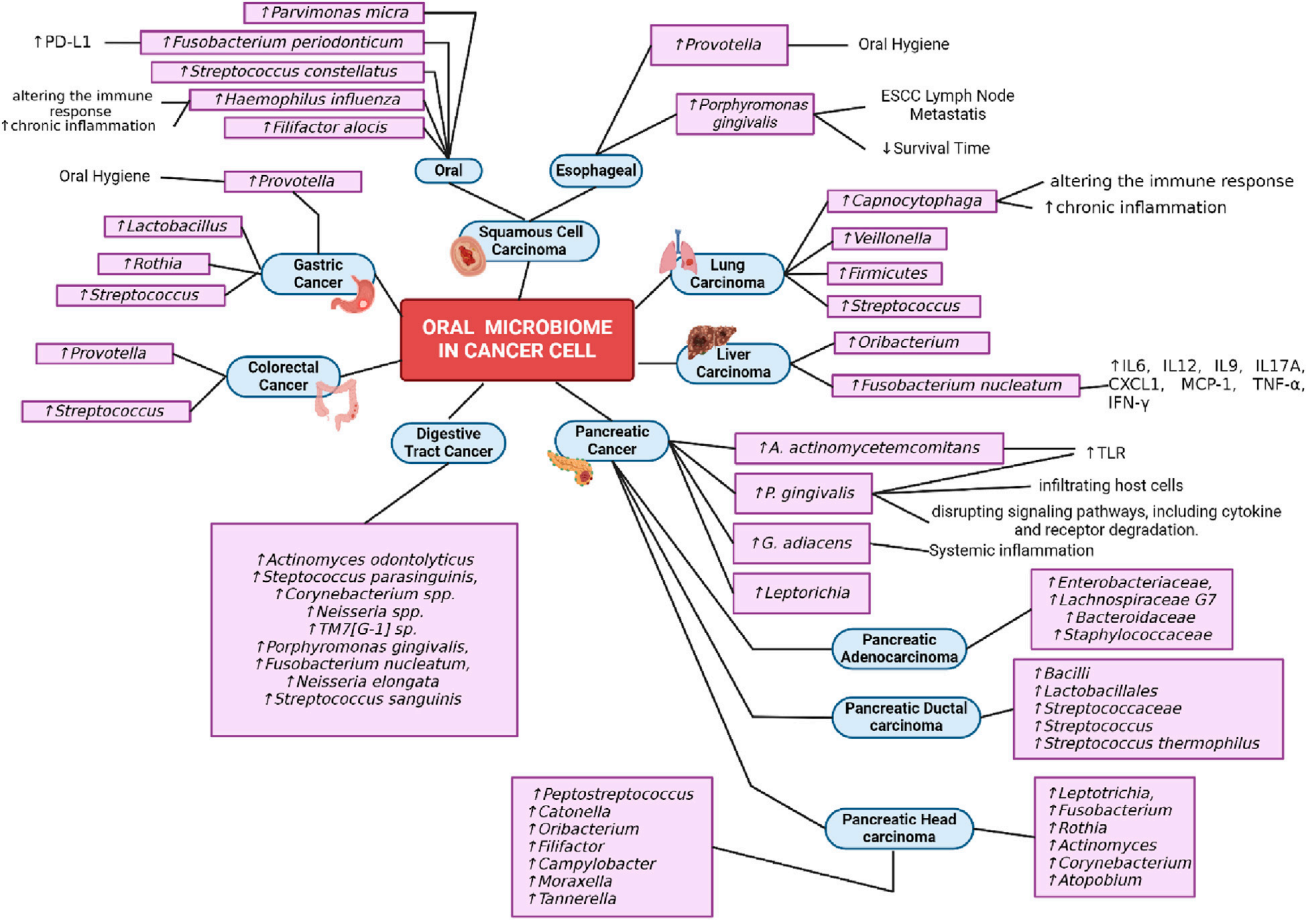
Oral Microbiome in Cancer. Created with BioRender.com Premium License by Fahrul Nurkolis (https://app.biorender.com, accessed on 10th July 2024).

In study by Kageyama et al. (2019) found that in patient with digestive tract cancer increase *Actinomyces odontolyticus, Steptococcus parasinguinis, Corynebacterium spp., Neisseria spp.,TM7[G-1] sp., Porphyromonas gingivalis, Fusobacterium nucleatum, Neisseria elongata* and *Streptococcus sanguinis* than the healthy patients (Table 9) (Kageyama et al., 2019). In study by Sun et al. (2018) mentioned *H. pylori* one of the strongest risk of gastric cancer. *H. pylori* infection triggers an innate immune response, leading to increased oxidative stress (Figure 4). This can cause DNA damage and promote carcinogenesis (Sun et al., 2018).

**TABLE 9:** Oral microbiota related to development of cancer.

No	Studies	Outcomes	References
1	Investigated oral microbiome composisition from mouthwash samples in esophageal adenocarcinoma (EAC) (n = 81) and esophageal squamos cell carcinoma (ESCC) (n = 25) using 16S rRNA gene sequencing	- Periodontal pathogen *Tannerella forysthia* higher risk of EAC - Commensal genus *Neisseria* and *Streptococcus pneumoniae* lower EAC risk - Increase *Porphyromonas* gingivalis *in ESCC*	Peters et al. (2017)
2	Investigated oral microbiome associated with esophageal squamos cell carcinoma (ESCC) in fasting saliva samples were collected ESCC case (n = 87), dysplasia (n = 63), and healthy controls (n = 85) using 16S rRNA sequencing	- Found that decrease carriage of genera *Lautropia*, *Bulleidia, Catonella, Corynebacterium, Moryella, Peptococcus* and *Cardiobacterium* in ESCC subject compared non-ESCC subject are significantly associated with an increase risk of ESCC - Higher of Prevotella and *Streptococcus* were also observed in the ESCC group compared to non-ESCC groups	Chen et al. (2015)
3	Investigated in case control study the relationship of oral microbiota with subsequent risk of pancreatic cancer from oral wash sampel adenocarcinoma pancreas (n = 361) and controls (n = 371) using bacterial 16S rRNA gene sequencing	- Oral pathogens *Porphyromonas gingivalis* and *Aggregatibacter actinomycetemcomitans* associated with higher risk of pancreatic cancer - Higher relative abundance of the phylum *Fusobacteria* and genus *Leptotrichia* associated decreased pancreatic cancer risk	Fan et al. (2018)
4	Cohort studies oral microbiota increase risk pancreatic cancer from saliva of patients with pancreatic cancer (n = 10) and healthy controls (n = 10) using real-time quantitative PCR (qPCR)	- The levels of *N. elongata and S. mitis* singnificantly decrease in patients with pancreatic cancer than healthy controls an ROC-plot AUC value of 0.90 with 96.4% sensitivity and 82.1% specificity - The level of *G. adiacens* significantly elevated in patients with pancreatic cancer relative to all non-cancer subjects an ROC-plot AUC value of 0.70 with 85.7% sensitivity and 55.6^ specificity	Farrell et al. (2012)
5	Characterization salivary microbiome in patients with pancreatic cancer (n = 8), other disease (n = 78), and healthy controls (n = 22) using 16S rRNA sequencing	- Found that significantly higher ratio of *Leptotrichia* to *Porphyromonas* in the saliva patients pacreatic cancer that healthy patients or patients with other disease (p < 0.0001) - Meanwhile, found that lower non significant relative abundances of *Neisseria* and *Aggregatibacter* in saliva of pancreatic cancer patients (p < 0.05)	Torres (2015)
6	Characterization oral microbiome in patients with pancreatic adenocarcinoma (n = 273) and healthy controls (n = 285) extracted from saliva samples using 16S rRNA gene was PCR amplified	- No association were detected for alpha diversity with pancreatic cancer also indication associations between specific taxa and pancreatic cancer - Increasing relative levels of *Haemophilus* associated with decreased odds of pancreatic cancer, while found of *Enterobacteriaceae, Lachnospiraceae G7, Bacteroidaceae* or *Staphylococcaceae* associated with increased odds of pancreatic cancer - Found the association between pancreatic cancer and the microbioal community composition (i.e., beta diversity)	Vogtmann et al. (2020)
7	Characterization oral microbiota in patients with pancreatic ductal adenocarcinoma (n = 40), intraductal papillary mucinous neoplasm (n = 39), and controls (n = 58) provided saliva sample surveyed by sequencing of the 16S rRNA microbial genes	- PDAC cases found higher levels of *Firmicutes* and related taxa (*Bacilli, Lactobacillales, Streptococcaceae, Streptococcus, Streptococcus thermophilus*) compared to controls - *Gammaproteobacteria, Pasteurellales, Pasteurellaceae, Haemophilus, Haemophilus parainfluenzae; and Betaproteobacteria, Neisseriales, Neisseriaceae, Neisseria, Neisseria flaviscens* has lower in PDAC but higher levels in controls - The PDAC and IPMN groups very similar in measures of alpha diversity of the oral microbiota	Olson et al. (2017)
8	Reveal the bacterial composisiton in the microbiota of tongue coating in pancreatic head carcinoma (n = 30) and healthy controls (n = 25) using 16S rRNA gene sequencing technology	- In patient with pancreatic head carcinoma overpresented microbiota than healty controls such as *Leptotrichia, Fusobacterium, Rothia, Actinomyces, Corynebacterium, Atopobium, Peptostreptococcus, Catonella, Oribacterium, Filifactor, Campylobacter, Moraxella* and *Tannerella* - Meanwhile in healthy controls increasing microbiome such as *Haemophilus, Porphyromonas*, and *Paraprevotella*	Lu et al. (2019)
9	Deep sequencing using 16S ribosomal RNA (rRNA) reveals microbiota dysbiosis of tongue coat in patient with liver carcinoma (n = 35) and healthy subjects (n = 25)	- *Fusobacterium* and *Oribacterium* increase in liver carcinoma than healthy subjects	Lu et al. (2016)
10	Variations of salivary microbiota association in patient with lung cancer ((n = 20) including squamos cell carcinoma (SCC) (n = 10) and adenocarcinoma (AC) (n = 10) and control subjects (n = 10) using 16S sequencing analysis	- Two bacterial including *Capnocytophaga* sp., *Veillonella* sp. distinguishing with SCC from control subjects ROC value 0.86 with 84.6% sensitivity and 86.7% specificity. In patients with AC distinguishing from control subjects with ROC value 0.80, sensitivity 78.6%, and specificity 80%	Yan et al. (2015)
11	Identified bacteria biomarkers associated with oral squamous cell carcinoma (OSCC) from salivary sample healthy individuals (n = 51) and oral squamous cell carcinoma (n = 197) using 16S rRNA squencing	- *Fusobacteria* increased significantly with the progression of oral cancer from the healthy controls (2.98%) to OSCC stage 1 (4.35%) through stage 4 (7.92%) - *Fusobacterium periodonticum, Parvimonas micra, Streptococcus constellatus, Haemophilus influenza, and Filifactor alocis* associated with OSCC, and progressively increased in abundance from stage 1 to stage 4	Yang et al. (2018)
12	Revealed salivary microbiome compositions in patients from non-small cell lung cancer (NSCLC) (n = 39) compared with healthy controls (n = 20) using 16S rRNA sequencing	- Phylum *Firmicutes* and two genera *Streptococcus* and *Veillonella* increase in NSCLC patients compared with controls	Zhang et al. (2019)
13	Case-control study oral rinse DNA sample from 190 patient with colorectal cancer used to amplify V3-V4 region of bacterial 16S rRNA gene	- Increased genus of *Lactobacillus* and *Rothia* in patient with cancer colorectal	Kato et al. (2016)
14	Investigated alteration oral microbiome linked with colorectal cancer from oral swabs, colonic mucosae and stool in individuals with colorectal cancer (n = 99), colorectal polyps (n = 32), and controls (n = 103) than sequencing using 16S rRNA gene amplicon	- An increase several ral taxa was found in colorectal cancer compared with control such as *Streptococcus* and *Prevotella* spp.	Flemer et al. (2018)
15	Case-control study examined the salivary microbiome in patients with digestive tract cancer (n = 59) and control subjects (n = 118) then sequencing using 16S rRNA gene	- *Actinomyces odontolyticus, Steptococcus parasinguinis, Corynebacterium spp., Neisseria* s*spp.,TM7[G-1] sp., Porphyromonas gingivalis, Fusobacterium nucleatum, Neisseria elongata* and *Streptococcus sanguinis* was more abundant in the saliva of digestive tract cancer compared with in control subjects	Kageyama et al. (2019)
16	Investigate the characteristics of oral microbiome in gastric cancer from plaques and saliva samples including individuals with gastric cancer (n = 37) and controls (n-13) then sequencing by 16S rRNA gene amplicons	- Overall increased microbial diversity in cancer patients - Oral bacteria are more complex in patients with gastric cancer than the control populations - One of the strongest risk factors for gastric cancer is detection rate of *H*. *pylori*	Sun et al. (2018)

## 5 Skin microbiome and aging-related diseases

### 5.1 Diabetes

An adverse relationship was observed between the microbiome diversity and saliva levels in the skin of individuals with diabetes (Thimmappaiah Jagadeesh et al., 2017; Pang et al., 2020). The rise in diversity is a result of higher glucose levels in the perspiration of individuals with diabetes. Diabetes patients commonly experience thermoregulatory dysfunction caused by neuropathy (Thimmappaiah Jagadeesh et al., 2017). The diversity of the group varies dynamically as the disease progresses, with the group that has had diabetes for the longest duration exhibiting the highest level of diversity (Pang et al., 2020).

Furthermore, the diabetic group exhibited a notably increased prevalence of *S. epidermidis* in addition to their diversity (Table 10). This suggests excessive proliferation, which serves as the initial phase of infiltration into aseptic tissue. This rise in incidence may indicate a transition from harmless microorganisms to disease-causing agents as a result of extensive colonization. In addition to *S. Epidermidis*, there were also observed alterations in the composition of *Bacillus SPP* and *Staphylococcus aureus*, however these changes were not deemed to be significant (Thimmappaiah Jagadeesh et al., 2017).

**TABLE 10:** Skin microbiota related to development of diabetes.

No	Studies	Outcomes	References
1	Observe and compare the skin microbiome on the feet in 4 groups, healthy patients, diabetes sufferers <2 years, diabetes sufferers 5–8 years, diabetes sufferers >10 years	• The diversity in the foot of diabetic patients progressively rises with time in correlation with the length of the condition, when compared to control subjects • The foot skin microbiome of diabetes patients throughout a 2-year timeframe is primarily composed of Parvarchaeota, Chloroflexi, Euryarchaeota, Gemmatimonadetes, OP11, OD1, Elusimicrobia, TM6, Planctomycetes, OP3, and Firmicutes • The foot skin microbiome of diabetes patients with a duration of 5–8 years is primarily composed of Actinobacteria, Armatimonadetes, Verrucomicrobia, Cyanobacteria, Thermi, and Tenericutes • The foot skin microbiome of diabetes individuals who have had the condition for more than 10 years is primarily composed of Nitrospirae, Fusobacteria, Thermotogae, Aquificae, SR1, Chlamydiae, Spirochaetes, WPS-2, Acidobacteria, Crenarchaeota, and Proteobacteria	Pang et al. (2020)
2	Observational and case controlled study	• *S. epidermidis* was significantly more prevalent in the diabetic group compared to the non-diabetic group (77.5% vs. 53.7% of samples) • *Bacillus* species were significantly less prevalent in the diabetes group compared to non-diabetes (15.0% vs. 34.1% of samples) • The growth of *S*. *epidermidis* measured by CFU was significantly higher in the diabetic group than in the non-diabetic group • Patients with T2D are 5.40 times more at risk of experiencing heavy colonization from s epidermidis	Thimmappaiah Jagadeesh et al. (2017)

### 5.2 Cancer

There is a study that examines microbiome changes in cancer (Table 11). In skin cancer patients, the environment becomes unfavorable for *Cutibacterium acnes*. Cancerous skin experiences a reduction in sebaceous glands and leads to drier skin, which is detrimental to *C. acnes* and beneficial for *S. aureus*. This change leads to a decrease in *C. acnes*, reducing its protective effect against *S. aureus* resulting in colonization that leads to a pro-inflammatory environment. Therefore, skin cancer patients are characterized by increased *S. aureus* and a decrease in *C. acnes* (Voigt et al., 2022). Antoher study conducted by Ding et al. found that increased *S. aureus* prevalence with decline in commensal organisms is seen in squamous cell carcinoma and actinic keratosis, compared to healthy skin. While the microbiome of melanoma appears to be distinct from healthy skin, limited data is available to draw meaningful conclusions. Our review summarizes the current evidence on the microbiome of keratinocyte skin cancers and melanoma. The study establishes that the microbiome of these cancers is altered from healthy skin and that this dysbiosis involves both pathogenic and commensal organisms (Ding et al., 2024). It is also possible that the consumption of plants that have antimicrobial properties also plays a role in modulating aging-related diseases (Abbas et al., 2023).

**TABLE 11:** Skin microbiota related to development of cancer.

No	Studies	Outcomes	References
1	Investigating skin microbiome in squamos cell carcinoma (SCC) and premalignant actinic keratosis compared with healthy skin to identify skin cancer-associated changes in the skin microbiome from skin swabs. Overall 336 samples including patient with healthy skin (n = 138), actinic keratosis (n = 76), SCC (n = 93) and the controls patient with healthy skin (n = 29)	• For instance, the pathobiont *Staphylococcus aureus* was found to be increased at the expense of the commensal *Cutibacterium acnes* in SCC compared to healthy skin • The most discriminant genera were *Cutibacterium* (healthy skin-associated) and *Staphylococcus* (AK- and SCC-associated) • At the species level, multiple *Lactobacillus* species (e.g., L. *rhamnosus* and L. *plantarum*) were less abundant in AK and SCC, whereas *Ralstonia*, particularly *R. pickettii*, was positively associated with AK and SCC	Voigt et al. (2022)

## 6 Future implications and strategies

Recent developments in metagenomic analysis have simplified microbiota analysis. Although the metabolic implications of oral microbial profiles were similar to those of the gut microbiome, albeit at a lesser level, they may nevertheless play a significant role as biodetectors of diseases, especially its potential for the oral microbiota as a potential skin cancer biomarker and aging-genetic diseases. More study is needed to determine the accuracy and precision of salivary biomarkers, which are essential for the early identification of disease. Validation of combination biomarkers is necessary to increase saliva detection accuracy. More effective validation will result from improved identifying techniques. Due to differences in marker levels, salivary biomarkers need to be corrected for total salivary proteins. Research to date has concentrated on broad, readily recognizable factors found in bodily fluids and illness phases.

Majority of the study found out that Proteins, metabolites, changes to DNA, and miRNA are salivary biomarkers of aging disease, especially of Alzheimer’s disease that may be employed in non-invasive diagnostic procedures for early identification and primary prevention. Protein biomarkers in a non-invasive biological sample of the aged population may be found in saliva. The associated aging-disease, saliva is a potentially useful and universal diagnostic fluid substitute for Alzheimer’s disease that may help with early detection and treatment. Not just predict the disease, in the future, saliva may be used to identify persons with increased vulnerability to it, and locate active disease locations.

The saliva need a further studies which are -salivary AGE and 8-OHdG have good diagnostic usefulness in evaluating the aging process. Novel biomarkers, species, and salivary amyloid-β are promising diagnostic tools for Parkinson’s and Alzheimer’s diseases. Sirtuins contains of SIRT1, SIRT3, and SIRT6 in saliva can be an extra technique for non-invasive intravital diagnosis of Alzheimer’s disease in elderly individuals. The salivary GSH:GSSG ratio demonstrate lower levels in the elderly did not achieve a substantial level of relevance for usage as an aging predictor that require more research. By providing means of assessing the health of both well and patients, salivary biomarkers have the potential to decrease invasive medical procedures and increase noninvasive disease detection.

As an important note, for several diseases such as Alzheimer’s disease, Parkinson’s disease, heart failure, COPD, AMD, BPH, and atherosclerosis, there are no studies that discuss the skin microbiome and its relationship with these diseases, only papulopustular rosacea and its relationship with systemic disease (atherosclerosis); So exploratory studies are needed regarding the relationship between the skin microbiome and these diseases and this is a novelty that can be carried out by researchers. Future research should explore the relationship between skin and oral/salivary microbiome and telomere length, and its relationship to insulin sensitivity.

21st-century omics technologies, such as metagenomics, metabolomics, and transcriptomics, play a significant role in advancing research on microbiome detection for various diseases. These cutting-edge techniques enable comprehensive analysis of microbial communities, revealing their complex compositions and interactions within different environments. Metagenomics allows researchers to sequence and characterize the genetic material of entire microbial populations, providing insights into their functional capabilities and potential roles in disease processes. Metabolomics helps identify metabolites produced by these microbes, linking specific metabolic profiles to health outcomes. Additionally, transcriptomics can uncover gene expression patterns that may be altered in disease states, shedding light on microbial responses to host factors. Together, these omics technologies enhance our understanding of the microbiome’s role in health and disease, paving the way for innovative diagnostics and personalized treatment strategies.

21st-century omics technologies are crucial for analyzing the skin and salivary microbiome, as they provide detailed insights into microbial diversity and function in these specific environments. For the skin microbiome, techniques like metagenomics allow researchers to identify and characterize the vast array of microorganisms residing on the skin’s surface, linking shifts in microbial populations to conditions such as eczema, acne, and other dermatological issues. Similarly, in salivary microbiome analysis, these technologies can uncover the relationship between oral microbes and systemic diseases, including periodontal disease and even cardiovascular conditions. By utilizing metabolomics, researchers can detect specific metabolites produced by these microbiomes, offering valuable biomarkers for health monitoring. Overall, omics technologies enhance our understanding of how the skin and salivary microbiomes influence health and disease, facilitating the development of targeted interventions and personalized healthcare strategies.

## 7 Conclusion

Aging is a natural process influenced by genetic, environmental, and lifestyle factors, leading to age-associated diseases that account for a significant number of global deaths. The mechanisms of aging involve cellular senescence, impacting tissue regeneration and repair, with the senescence-associated secretory phenotype playing a crucial role in communication with immune cells and potentially contributing to inflammation and disease in older individuals. The aging process also leads to changes in the immune system and metabolism, increasing the risk of microbiome diversity in the body, which can manifest in various diseases. This highlights the potential of salivary and skin microbiomes in detecting aging-related changes and diseases, emphasizing the importance of further research in utilizing them as disease detectors for aging-associated conditions.

The human microbiome consists of microorganisms that reside in specific anatomical sites and interact with the human body, creating a distinct ecosystem. Throughout a person’s lifetime, the microbiome undergoes continuous evolution in response to changes in the human body, which can shift the balance of interactions and predispose individuals to various diseases. The oral cavity and saliva harbor a diverse microbial community that plays a crucial role in maintaining oral health, while the skin microbiome, consisting of bacteria, fungi, viruses, and mites, serves as a protective barrier against harmful pathogens. Changes in the oral and skin microbiomes can lead to various diseases, emphasizing the importance of maintaining a balanced microbial community for overall health and well-being.

Various aging-related diseases, such as Alzheimer’s, Parkinson’s, heart failure, atherosclerosis, Type 2 diabetes, Non-alcoholic fatty liver disease, osteoarthritis, osteoporosis, COPD, BPH, AMD, and cancer, have distinct pathogenic mechanisms influenced by aging. Understanding these mechanisms is crucial for addressing these conditions to improve overall health and well-being. For example, Alzheimer’s disease is characterized by neuronal death and DNA methylation alterations, while Parkinson’s disease involves mitochondrial dysfunction and protein degradation impairment. Conditions like atherosclerosis and diabetes are linked to inflammation and metabolic changes, highlighting the multifactorial nature of these diseases and the importance of early detection and personalized interventions.

The significant connection between the oral microbiome and various health conditions, including Alzheimer’s disease, Parkinson’s disease, diabetes, NAFLD, MAFLD, osteoarthritis, and cancer, underscores the importance of oral health and microbiome composition in managing and potentially diagnosing these diseases. Additionally, skin microbiome diversity and composition can be influenced by conditions like diabetes, potentially impacting the risk of developing skin cancer. Research indicates strong correlations between microbiome changes and various diseases, highlighting the potential of microbiome analysis in early detection and personalized treatment strategies. From these insights, it is evident that the salivary and skin microbiome hold significant potential as biodetectors for aging-associated diseases, warranting further investigation and validation in clinical settings.
